# ﻿Two new species and a new record of the genus *Nephrotoma* Meigen, 1803 (Diptera, Tipulidae) from Northwest China, with a key to species in the region

**DOI:** 10.3897/zookeys.1251.144156

**Published:** 2025-09-05

**Authors:** Xue Wang, Jinlong Ren, Ding Yang, Zhenning Chen

**Affiliations:** 1 Key Laboratory of the Pest Monitoring and Safety Control on the Crop and Forest, College of Agronomy, Xinjiang Agricultural University, Urumqi 830052, China Xinjiang Agricultural University Urumqi China; 2 Department of Entomology, China Agricultural University, Beijing 100193, China China Agricultural University Beijing China; 3 Key Laboratory of Biodiversity Formation Mechanismand Comprehensive Utilization of Qinghai-Tibetan Plateau in Qinghai Province / School of Life Science, Qinghai Normal University, Xining, Qinghai 810008, China Qinghai Normal University Xining China

**Keywords:** Key, Qinghai, taxonomy, Tipulinae, Xinjiang

## Abstract

This study describes two new species of the genus *Nephrotoma* Meigen, 1803 from Northwest China, *N. furvligulata***sp. nov.** and *N. palacea***sp. nov.**, with full descriptions and illustrations. It also records *N. lunulicornis* (Schummel, 1833) for the first time in China. Updated descriptions and illustrations are also provided for three previously known species, *N. geniculata* Yang & Yang, 1987, *N. joneensis* Yang & Yang, 1990, and *N. ligulata* Alexander, 1925, incorporating new morphological details. An identification key to all *Nephrotoma* species currently recorded in Northwest China is included to facilitate further research.

## ﻿Introduction

The genus *Nephrotoma* Meigen, 1803 is one of the largest within the family Tipulidae, with 485 known species distributed globally. These species are distributed as follows: 167 taxa in the Palaearctic Region, 40 in the Nearctic Region, 30 in the Neotropical Region, 125 in the Afrotropical Region, 129 in the Oriental Region, and 27 in the Australasian/Oceanian Region (Oosterbroek 2024). To date, 101 species have been identified in China. The genus is characterized by the several distinctive features: the body is typically yellow with dark stripes on the thorax; the *Rs* vein is short, and the cell m1 is usually sessile but can occasionally be petiolate; in males, tergite 9 is separated from sternite 9 and varies in shape, with the posterior margin of tergite 9 covered with tiny black spines; the lobe of the gonostylus is generally fleshy, flattened, and more or less acuminate; female cerci are longer than the hypogynial valves, typically blunt at the apex but sometimes acute, the valves may be tapered or parallel-sided (Oosterbroek 1978; Tangelder 1984; Ren and Yang 2017).

Northwest China encompasses six provinces and autonomous regions: Xinjiang, Shaanxi, Ningxia, Gansu, Qinghai, and the western part of Inner Mongolia. Until now, 29 species and one subspecies of the genus *Nephrotoma* were known to occur in this region (Oosterbroek 2024). A review of existing literature revealed that for *N. geniculata* Yang & Yang, 1987, *N. joneensis* Yang & Yang, 1990 and *N. ligulata* Alexander, 1925 descriptions and photographs were insufficient. Consequently, this study provides redescriptions and additional photographs for these species. Furthermore, we describe two new species of *Nephrotoma*, *N. furvligulata* sp. nov. and *N. palacea* sp. nov., providing detailed descriptions and illustrations. Additionally, we report the first record of *N. lunulicornis* (Schummel, 1833) from China. An identification key for the *Nephrotoma* species found in Northwest China is provided.

## ﻿Materials and methods

The specimens were collected in Xinjiang and Qinghai, China, during July and August from 2016 to 2023. They were examined using a Shunyu SZN45 stereomicroscope. Photographs were taken with a Nikon D750 camera equipped with a Canon MP-E 65 mm macro lens for habitus and wings and a Nikon ECLIPSE Ni upright microscope for detailed structures such as the semen pump and ovipositor. Image stacking was performed using Adobe Photoshop 2023 (Adobe Systems Ltd). Specimen descriptions were verified by examining individuals immersed in 75% ethyl alcohol (C_2_H_5_OH). Male genitalia were examined after macerating the apical portion of the abdomen in hot 10% NaOH for 8–15 min. The studied specimens are deposited in the collections of the Entomological Museum of China Agricultural University (**CAU**), Beijing, China, and Xinjiang Agricultural University (**XJAU**), Xinjiang, China.

The morphological terminology mainly follows Cumming and Wood (2017), de Jong (2017) for wing venation and Ribeiro (2006) for the lobe of gonostylus and clasper of gonostylus. The following abbreviations in figures are used: **A** = anal vein, **aia** = anterior immovable apodeme, **ba** = bifurcated appendage of sternite 8, **bk** = beak, **C** = costa, **ca** = compressor apodeme, **ce** = cercus, **cg** = clasper of gonostylus, **Cu** = cubitus, **d** = discal cell, **d ct** = dorsal crest, **gcx** = gonocoxite, **hy** = hypovalve, **lg** = lobe of gonostylus, **l bk** = lower beak, **o b lb** = outer basal lobe, **p ct** = posterior crest, **M** = medius, **pia** = posterior immovable apodeme, **R** = radius, **Sc** = subcosta, **sp** = sclerotized projection of sternite 9, **st 8** = sternite 8, **st 9** = sternite 9, **tg 8** = tergite 8, **tg 9** = tergite 9, **tg 10** = tergite 10.

## ﻿Taxonomy

### ﻿Checklist of *Nephrotoma* crane flies from Northwest China

*Nephrotoma aculeata* (Loew, 1870)

*Nephrotoma analis* (Schummel, 1833)

*Nephrotoma appendiculata appendiculata* (Pierre, 1920)

*Nephrotoma barbigera* (Savchenko, 1964)

*Nephrotoma basiflava* Yang & Yang, 1991

*Nephrotoma concava* Yang & Yang, 1990

*Nephrotoma cornicina cornicina* (Linnaeus, 1758)

*Nephrotoma drakanae* Alexander, 1936

*Nephrotoma furvligulata* sp. nov.

*Nephrotoma geniculata* Yang & Yang, 1987

*Nephrotoma hirsuticauda* Alexander, 1924

*Nephrotoma hubeiensis* Yang & Yang, 1987

*Nephrotoma hypogyna* Yang & Yang, 1990

*Nephrotoma joneensis* Yang & Yang, 1990

*Nephrotoma kanasensis* Ren & Yang, 2017

*Nephrotoma koreana* Tangelder, 1984

*Nephrotoma ligulata* Alexander, 1925

*Nephrotoma lundbecki lundbecki* (Nielsen, 1907)

*Nephrotoma lunulicornis* (Schummel, 1833)

*Nephrotoma martynovi* Alexander, 1935

*Nephrotoma palacea* sp. nov.

*Nephrotoma parvinotata* (Brunetti, 1918)

*Nephrotoma parvirostra* Alexander, 1924

*Nephrotoma perobliqua* Alexander, 1936

*Nephrotoma pjotri* Tangelder, 1984

*Nephrotoma profunda* Alexander, 1935

*Nephrotoma qinghaiensis nigrabdomen* Yang & Yang, 1990

*Nephrotoma qinghaiensis qinghaiensis* Yang & Yang, 1987

*Nephrotoma quadristriata* (Schummel, 1833)

*Nephrotoma scurra* (Meigen, 1818)

*Nephrotoma sinensis* (Edwards, 1916)

*Nephrotoma tenuipes* (Riedel, 1910)

*Nephrotoma xinjiangensis* Yang & Yang, 1987

### ﻿Key to species (males) of *Nephrotoma* from Northwest China

Updated from Ren and Yang 2017.

**Table:** 

1	Body mainly black	**2**
–	Body mainly yellow	**4**
2	Vertex with tubercle prominent (Fig. 30); pronotum dark yellow (Fig. 31)	***Nephrotoma joneensis***
–	Vertex with tubercle low or absent (Fig. 69); pronotum mainly black (Fig. 70)	**3**
3	Vertex with a distinct yellow band around the posterior margin of the eye; tergite 9 posteriorly with four projections (Yang and Yang 1991: fig. 3, 2002: fig. 1); sternite 8 with posterior margin straight, without appendage (Yang and Yang 1991: fig. 4, 2002: fig. 2)	***Nephrotoma basiflava***
–	Vertex without the above characteristics (Fig. 70); tergite 9 posteriorly with two projections (Fig. 74); sternite 8 posteriorly sunken at the middle, with a horned, hairy, and sclerotized appendage (Fig. 75)	***Nephrotoma palacea* sp. nov.**
4	Sternite 8 with a median appendage (Figs 6, 20, 48)	**5**
–	Sternite 8 without median appendage (Fig. 62)	**19**
5	Prescutum and presutural scutum with lateral stripe straight (Fig. 59)	**6**
–	Prescutum and presutural scutum with lateral stripe bend outward (Figs 3, 17, 45)	**7**
6	Pronotum with small projection at the middle; sternite 8 posteriorly with an appendage directed ventrally; lobe of gonostylus narrow (Yang and Yang 1987a: fig. 2A)	***Nephrotoma qinghaiensis qinghaiensis***
–	Pronotum without the above characteristics (Ren and Yang 2017: fig. 2); sternite 8 posteriorly with an appendage directed caudally (Oosterbroek 1978: figs 89, 96A); lobe of gonostylus broad (Oosterbroek 1978: fig. 92)	***Nephrotoma aculeata***
7	Wing tip with numerous small macrotrichiae (Ren and Yang 2017: fig. 41)	***Nephrotoma tenuipes***
–	Wing tip bare	**8**
8	Abdomen brown (Yang and Yang 1990)	***Nephrotoma qinghaiensis nigrabdomen***
–	Abdomen yellow	**9**
9	Sternite 8 posteriorly with an appendage directed ventrally (Fig. 19)	**10**
–	Sternite 8 posteriorly with an appendage directed caudally (Figs 5, 47)	**12**
10	Lateral projections of tergite 9 shorter than the median projections (Yang and Yang 1990: fig. 2)	***Nephrotoma hypogyna***
–	Lateral projections of tergite 9 longer than the median projections (Fig. 21)	**11**
11	Tergite 9 with lateral lobes distinct, nearly triangular (Yang and Yang 1987a: fig. 6B)	***Nephrotoma parvirostra***
–	Tergite 9 with lateral lobes small, rounded (Fig. 21)	***Nephrotoma geniculata***
12	Sternite 8 posteriorly with a blunt appendage at the middle (Figs 6, 48)	**13**
–	Sternite 8 posteriorly with a sharp appendage at the middle (Ren and Yang 2017: figs 34, 35)	**17**
13	Sternite 8 with a median appendage bifid at apex (Figs 6, 48)	**14**
–	Sternite 8 with median appendage not bifid at apex (Oosterbroek 1978: figs 72, 160A, B; Yang and Yang 1987b: fig. 1A)	**15**
14	Space between lateral and median stripes on prescutum and presutural scutum broad (Fig. 45); mediotergite with median stripe thin (Fig. 45); abdominal tergites with thin longitudinal spots (Fig. 44)	***Nephrotoma ligulata***
–	Space between lateral and median stripes on prescutum and presutural scutum narrow (Fig. 3); mediotergite with median stripe broad (Fig. 3); abdominal tergites with broad longitudinal stripes (Fig. 2)	***Nephrotoma furvligulata* sp. nov.**
15	First flagellar segment yellow	***Nephrotoma hubeiensis***
–	First flagellar segment dark brown (Oosterbroek 1978: figs 72, 160A, B)	**16**
16	Occipital marking oval (Oosterbroek 1978: fig. 156A, B); sternite 8 posteriorly with a rod-like appendage at the middle (Oosterbroek 1978: fig. 160A, B); lobe of gonostylus posteriorly strongly sclerotized (Oosterbroek 1978: fig. 158)	***Nephrotoma cornicina cornicina***
–	Occipital marking sword-shaped (Oosterbroek 1978: fig. 66); sternite 8 posteriorly with a cylindrical-shaped appendage at the middle (Oosterbroek 1978: fig. 72); lobe of gonostylus without sclerotized (Oosterbroek 1978: fig. 68)	***Nephrotoma appendiculata appendiculata***
17	Tergite 9 posteriorly with two projections on each side (Ren and Yang 2017: fig. 36)	***Nephrotoma lundbecki lundbecki***
–	Tergite 9 posteriorly with one projection on each side (Yang and Yang 1987a: fig. 3B, 1990: fig. 3.2)	**18**
18	Tergite 9 posteriorly with V-notch at the middle (Yang and Yang 1987a: fig. 3B); sternite 8 posteriorly with long projection (Yang and Yang 1987a: fig. 3A)	***Nephrotoma xinjiangensis***
–	Tergite 9 posteriorly with U-notch at the middle (Yang and Yang 1990: fig. 3.2); sternite 8 posteriorly with short projection (Yang and Yang 1990: fig. 3.1)	***Nephrotoma concava***
19	Tergite 9 posteriorly with small projection on each side (Yang and Yang 1987a: fig. 6B; Liu 2011: fig. 7–69b; Ren and Yang 2017: fig. 9)	**20**
–	Tergite 9 posteriorly without projection on each side	**25**
20	Prescutum and presutural scutum with lateral stripe curved outward (Yang and Yang 1987a)	***Nephrotoma parvinotata***
–	Prescutum and presutural scutum with lateral stripe straight	**21**
21	Sternite 9 without ventral projection (Ren and Yang 2017: fig. 7)	***Nephrotoma analis***
–	Sternite 9 with ventral projection (Oosterbroek 1979: fig. 20; Tangelder 1984: figs 16, 149; Liu 2011: figs 7–69a)	**22**
22	Clasper of gonostylus without a crest (Liu 2011: figs 7–69c)	***Nephrotoma martynovi***
–	Clasper of gonostylus with a crest (Fig. 64; Oosterbroek 1979: fig. 22; Tangelder 1984: figs 20, 152)	**23**
23	Sternite 8 shallowly concave (Tangelder 1984: fig. 28)	***Nephrotoma pjotri***
–	Sternite 8 deeply concave (Tangelder 1984: figs 127, 150)	**24**
24	Lateral projections of tergite 9 shorter than the median projections (Tangelder 1984: fig. 151); sternite 9 intermediate appendage directed caudally (Tangelder 1984: fig. 149); sternite 8 posteriorly with narrow V-notch at the middle (Tangelder 1984: fig. 150)	***Nephrotoma koreana***
–	Lateral projections of tergite 9 longer than the median projections (Tangelder 1984: fig. 129); sternite 9 intermediate apendance directed ventrally (Oosterbroek 1979: fig. 20); sternite 8 posteriorly with broad V-notch at the middle (Tangelder 1984: fig. 127)	***Nephrotoma lunulicornis***
25	Prescutum and presutural scutum with lateral stripe straight	**26**
–	Prescutum and presutural scutum with lateral stripe curved outward	**31**
26	Prescutum and presutural scutum lateral stripe with a separate spot; anterior wing margin darker	***Nephrotoma sinensis***
–	Prescutum and presutural scutum lateral stripe without separate spot; anterior wing margin brighter	**27**
27	Mediotergite black	***Nephrotoma perobliqua***
–	Mediotergite yellow	**28**
28	Clasper of gonostylus without a crest (Tangelder 1984: fig. 261)	***Nephrotoma barbigera***
–	Clasper of gonostylus with a crest (Oosterbroek 1979: figs 11, 28; Tangelder 1984: figs 245, 271)	**29**
29	Occiput unmarked; sternite 8 without long bristles (Tangelder 1984: fig. 272)	***Nephrotoma profunda***
–	Occipital marking wedge-shaped; sternite 8 with long bristles (Oosterbroek 1979: figs 10, 25, 26)	**30**
30	Flagellomeres normal (Tangelder 1984: figs 253, 254); tergite 9 with large, outward-curving lateral projections (Oosterbroek 1979: fig. 27)	***Nephrotoma scurra***
–	Flagellomeres expanded bilaterally (Oosterbroek 1979: fig. 9a–g); tergite 9 without lateral projections (Oosterbroek 1979: fig. 12)	***Nephrotoma quadristriata***
31	Sternite 8 shallowly convex, with short setae (Ren and Yang 2017: fig. 19)	***Nephrotoma kanasensis***
–	Sternite 8 deeply concave, with long setae	**32**
32	Scape and pedicel dark brown	***Nephrotoma drakanae***
–	Scape and pedicel orange	***Nephrotoma hirsuticauda***

#### ﻿ Nephrotoma furvligulata sp. nov.

Taxon classificationAnimaliaDipteraTipulidae

37DD5B12-3633-5D7A-8DB4-2818B0D70B63

https://zoobank.org/29D9E450-4CCC-46D9-99FE-67CDF688ABB4

Figs 2–4, 5–9, 10–12, 13–15

##### Type material.

***Holotype*.** China – Qinghai Prov. • ♂; Qilian, Binggou; 3075 m a.s.l.; 38.1333°N, 100.1678°E; 27 Jul. 2023; X. Wang leg.; light trap; XJAU TN2310243.

**Figure 1.**
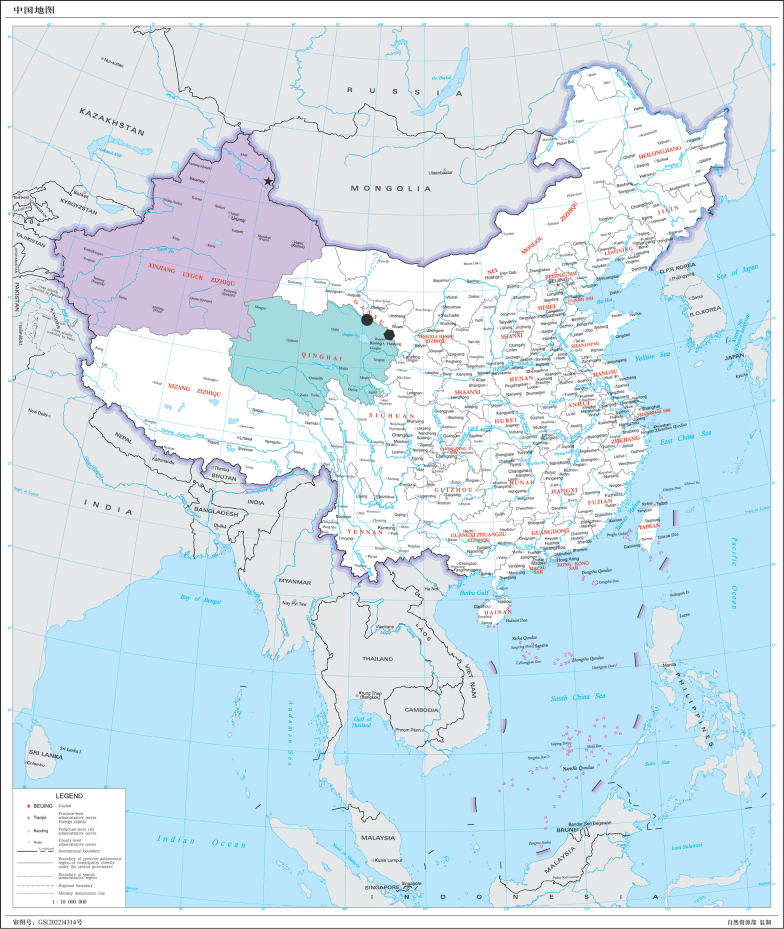
Distribution map showing collection sites of new *Nephrotoma* species and new record in China. Purple area indicates Xinjiang; cyan area indicate Qinghai; star indicates *N. palacea* sp. nov.; hexagon indicates *N. lunulicornis* (Schummel, 1833); circle indicates *N. furvligulata* sp. nov.

***Paratypes*.** China – Qinghai Prov. • 2 ♂♂, 3 ♀♀; Qilian, Lujiaogou; 2867 m a.s.l.; 38.1164°N, 100.3407°E; 30 Jul. 2023; X. Wang leg.; light trap; XJAU TN2310691 to TN231095 • 2 ♂♂, 2 ♀♀; Qilian, Lujiaogou; 3311 m a.s.l.; 38.1132°N, 100.4744°E; 30 Jul. 2023; X. Wang leg.; light trap; XJAU TN2310539 to TN2310542 • 3 ♂♂; Qilian, Huangcang Temple Water Conservancy Hub Project; 2579 m a.s.l.; 38.2499°N, 100.1836°E; 2 Aug. 2023; X. Wang leg.; light trap; XJAU TN2310734 to TN2310736.

##### Diagnosis.

Body yellow. Occipital marking triangular, dark brown, laterally with pale-brown spots. Antenna reaching the base of abdomen when bent backward. Space between lateral and median stripes of prescutum and presutural scutum narrow. Tergite 9 posteriorly with narrow median notch and broad, inclined projections, its margin covered with tiny black spines. Sternite 8 with a bifurcated appendage. Clasper of gonostylus with posterior crest possessing a triangular projection.

##### Description.

**Male.** Body length 9.0–11.1 mm, wing length 9.3–10.7 mm, antenna length 3.6–4.5 mm (*N* = 8).

**Figures 2–4.**
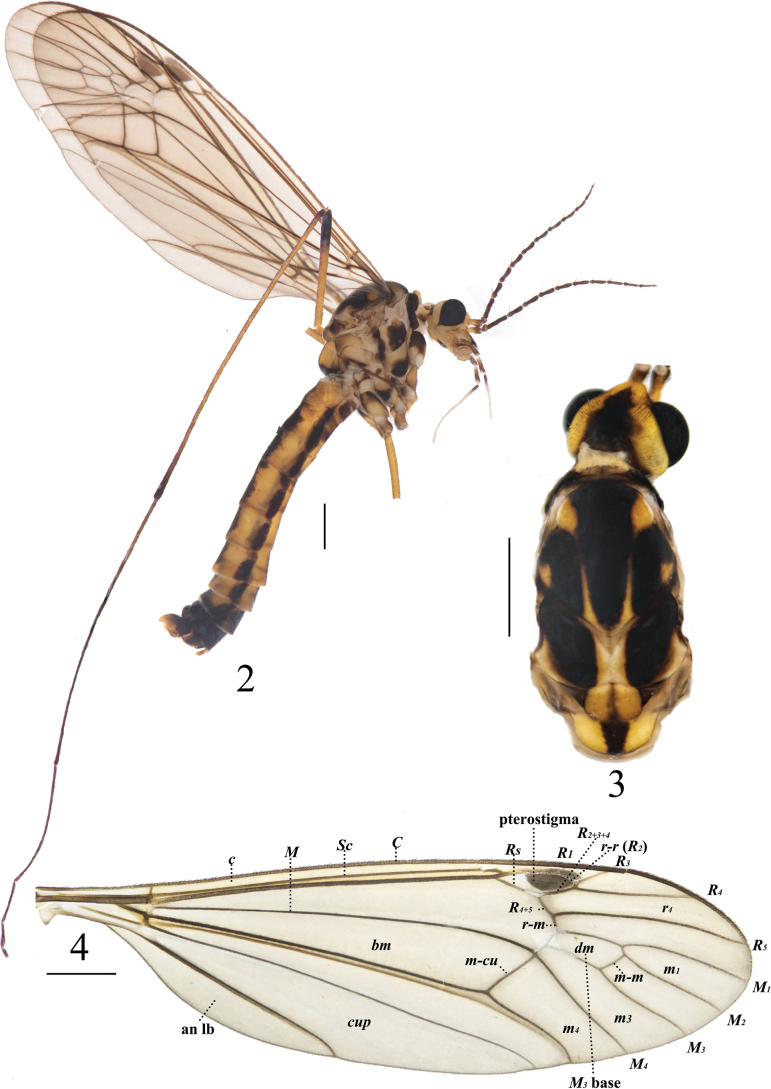
*Nephrotoma furvligulata* sp. nov., male. 2. Male habitus, lateral view; 3. Head and thorax, dorsal view; 4. Right wing. Scale bars: 1.0 mm.

***Head*** (Figs 2, 3). Mostly yellow, vertex with dark brown spot. Occipital marking triangular, dark brown, laterally with pale brown spots. Nasus dark brown. Rostrum brown. Head with brown setae. Antenna 13-segmented, reaching the base of abdomen when bent backward; scape yellow, with apical third brown, pedicel and flagellar segments brown. Length of verticils reaching almost 3/5 length of the corresponding segment. Terminal segment of flagellum round. Palpi brownish, terminal segment pale, darkened at base.

***Thorax*** (Figs 2, 3). Mostly yellow. Pronotum black, yellow middorsally. Prescutum and presutural scutum with three longitudinal black stripes; lateral stripe apically bent outward; space between lateral and median stripes narrow. Postsutural scutum yellow in the middle, each lobe with large black spot; posterior-lateral margin brown. Scutellum yellow with a black median line. Mediotergite yellow with broad black median stripe, with posterior margin blackened. Anepisternum and katepisternum basally each with a large black spot. Anepimeron blackened apically; katepimeron with small median black spot. Meron blackened basally. Legs yellow. Coxae yellow, variegated with dark brown spots at the base. Apices of tibiae and femora dark brown, tarsal segments dark brown. Wing pale brown with a distinct dark brown pterostigma and macrotrichiae; brown shadow at vein *R4+5* and crossvein *r-m*; cell *m* sessile (Fig. 4). Halter with stem dark brown; knob pale yellow with a dark brown margin (Fig. 2).

***Abdomen*** (Fig. 2). Mainly yellow. Abdominal tergites with three broad black longitudinal stripes; the median stripe broader than the lateral stripes. Abdominal sternites 5–7 with an interrupted longitudinal stripe. Abdominal segments 8–9 black.

**Figures 5–9.**
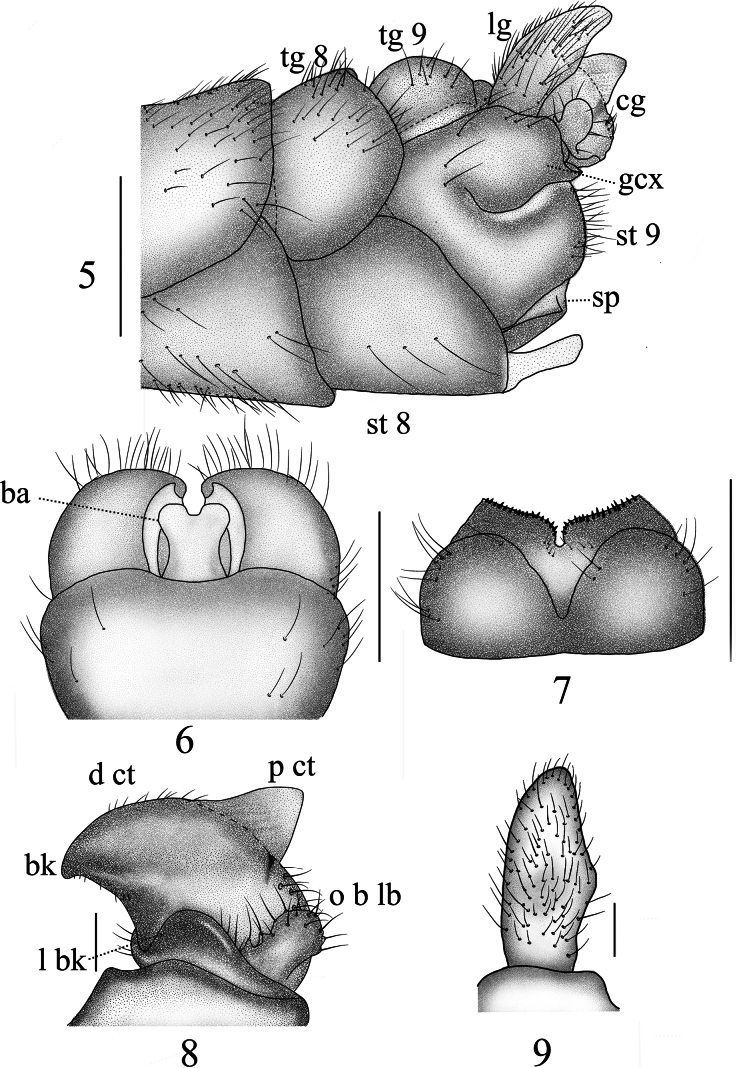
Male terminalia of *Nephrotoma furvligulata* sp. nov. 5. Hypopygium, lateral view; 6. Hypopygium, ventral view; 7. Tergite 9, dorsal view; 8. Clasper of gonostylus, lateral view; 9. Lobe of gonostylus, lateral view. Scale bars: 0.5 mm (5–7); 0.1 mm (8, 9).

**Figures 10–12.**
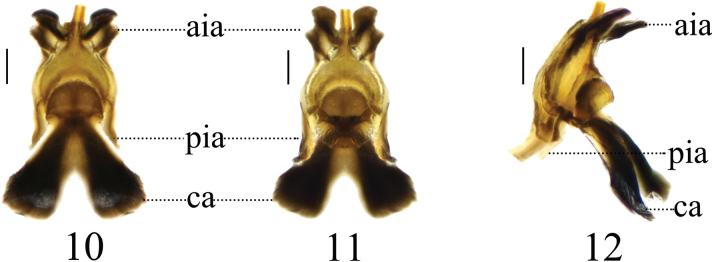
Semen pump of *Nephrotoma furvligulata* sp. nov. 10. Dorsal view; 11. Ventral view; 12. Lateral view. Scale bars: 0.1 mm.

***Hypopygium*** (Figs 5–9). Tergite 9 posteriorly with broad, inclined projections, its margin covered with tiny black spines (Fig. 7). Posterior margin with narrow, U-shaped notch. Sternite 8 apically with a bifurcated appendage (Fig. 6). Sternite 9 posteriorly with sparse setae, and a sclerotized projection at the middle (Figs 5, 6). Gonocoxite separated from sternite 9, irregularly oval with smooth and slightly curved margins, featuring a slightly indented inward apex (Fig. 5). Lobe of gonostylus flattened, with weak lateral protrusion and obtuse apex (Figs 5, 9). Clasper of gonostylus with a large concavity at the base; beak obtuse; posterior crest with a triangular projection and sparse setae; outer basal lobe nearly oval, apically rounded; lower beak with a vaulted protuberance; the margin extending from the beak to the lower beak, strongly sclerotized (Fig. 8). Semen pump with posterior immovable apodeme darkened, directed backward, tip outward (Figs 10–12). Compressor apodeme blackish-brown, fan-shaped, forming an 80° angle with the posterior immovable apodeme. Anterior immovable apodeme narrower than the compressor apodeme.

**Figures 13–15.**
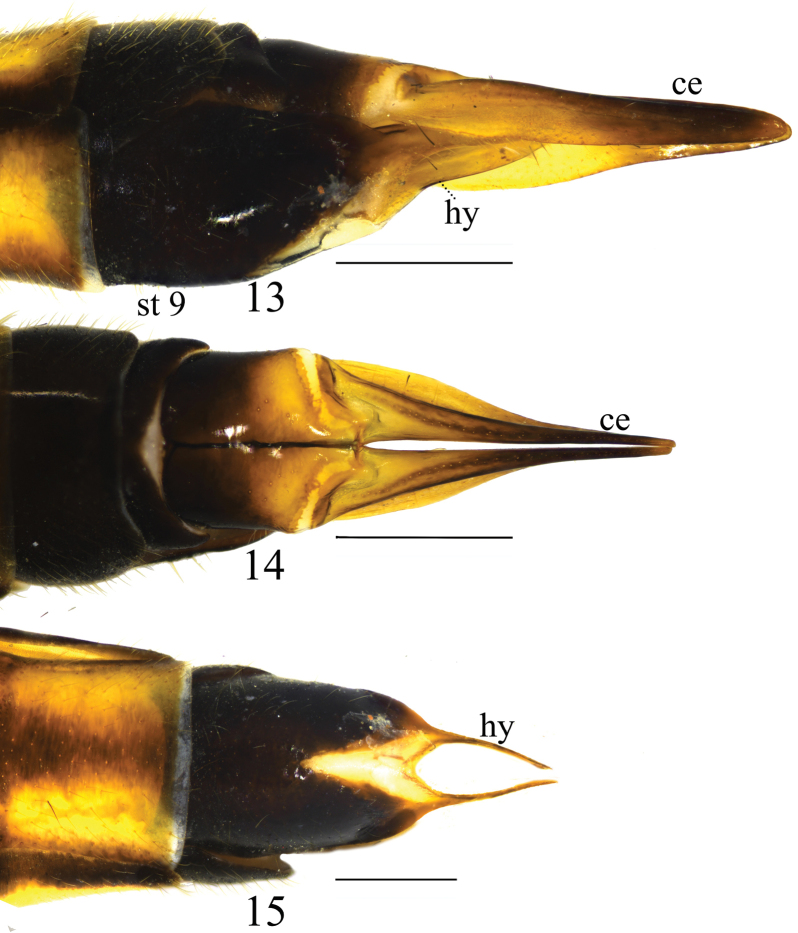
Female terminalia of *Nephrotoma furvligulata* sp. nov. 13. Ovipositor, lateral view; 14. Ovipositor, dorsal view; 15. Ovipositor, ventral view. Scale bars: 0.5 mm.

**Female.** Body length 12.9–15.1 mm, wing length 10.5–12.6 mm, antenna length 3.3–3.9 mm (*N* = 5). Resembles male in body coloration. Tergite 8, tergite 9, sternite 8, and basal region of tergite 10 dark brown (Figs 13–15).

***Ovipositor*** (Figs 13–15). Cercus narrowed toward tip (Figs 13, 15). Hypogynial valve curved upwards apically (Figs 13, 14).

##### Elevation range in China.

Adults were collected at altitudes ranging from 2000 m to 2500 m.

##### Period of activity.

Adults were collected only in late July.

##### Distribution.

China, Qinghai (Fig. 1).

##### Etymology.

The specific name (from Latin *furvus* (adj., meaning “dark”) and Latin *ligulatus* (adj., meaning “ligulate”)) refers to the abdominal tergites bearing a darker mid-longitudinal stripe and to sternite 8 posteriorly with a bifurcated appendage that extends outward at its apex in ventral view.

##### Remarks.

The new species is similar to *N. ligulata* Alexander, 1925, in the shape of sternite 8 and the lobe of the gonostylus. However, it can be distinguished by the following features: in *N. furvligulata* sp. nov. the space between the lateral stripe and the median stripe of the prescutum and presutural scutum is narrow; the mediotergite is yellow with a broad black median stripe; the abdominal tergites have broad longitudinal stripes. In *N. ligulata*, the space between the lateral stripe and the median stripe of the prescutum and presutural scutum is broad; the mediotergite is yellow with a thin black median stripe; the abdominal tergites on segments 1–4 have longitudinal stripes at the posterior margin (Mannheims 1967; de Jong 1993).

#### ﻿ Nephrotoma geniculata

Taxon classificationAnimaliaDipteraTipulidae

Yang & Yang, 1987

2E5CE128-A545-53A5-9264-85FB614709F1

Figs 16–18, 19–23, 24–26, 27–29

Nephrotoma geniculata Yang & Yang, 1987: 131.

##### Material examined.

China – Hubei Prov. • 1 ♂, 2 ♀♀; Shennongjiasongbai, 700–800 m a.s.l.; 23 Jun. 1984; X.L. Wang leg.; CAU Df000404, Df000405. – Qinghai Prov. • 1 ♂; Haidong, Shenba Village; 2455 m a.s.l.; 36.2496°N, 102.5274°E; 29 Jun. 2019; X. Li leg.; CAU TN2304237 • 7 ♂♂, 4 ♀♀; Menyuan, Sigou; 2489 m a.s.l.; 37.1362°N, 102.3763°E; 18 Jul. 2023; X. Wang leg.; light trap; XJAU TN2309123 to TN2309133 • 9 ♂♂, 4 ♀♀; Huzhu, Bazha Tibetan Autonomous Township; 2286 m a.s.l.; 36.9845°N, 102.4326°E; 18 Jul. 2023; X. Wang leg.; light trap; XJAU TN2309021 to TN2309033 • 1 ♂; Huzhu, Weibei Road; 2296 m a.s.l.; 36.9446°N, 102.4677°E; 21 Jul. 2023; X. Wang leg.; light trap; XJAU TN2309135 • 3 ♂♂, 2 ♀♀; Menyuan, Gangqing Road; 2370 m a.s.l.; 37.1008°N, 102.3439°E; 23 Jul. 2023; X. Wang leg.; light trap; XJAU TN2309259 to TN2309263 • 1 ♂, 1 ♀; Menyuan, Sigou; 2369 m a.s.l.; 37.1245°N, 102.3561°E; 23 Jul. 2023; X. Wang leg.; light trap; XJAU TN2309264 to TN2309265.

##### Diagnosis.

Body yellow. Occipital marking drop-shaped, apically pale brown. Antenna reaching the base of abdomen when bent backward. Wing pale brown, cell m1 petiolate. Tergite 9 posteriorly with four projections, median projections broad and covered with short, tiny black spines; lateral projections horn-shaped. Sternite 8 posteriorly with a bent, flattened protuberance, narrowing downwards in lateral view. Clasper of gonostylus with dorsal crest bearing a sclerotized and cristate projection.

##### Redescription.

**Male.** Body length 11.2–13.2 mm, wing length 12.1–13.2 mm, antenna length 4.7–5.1 mm (*N* = 23). Present description based on specimens preserved in alcohol.

**Figures 16–18.**
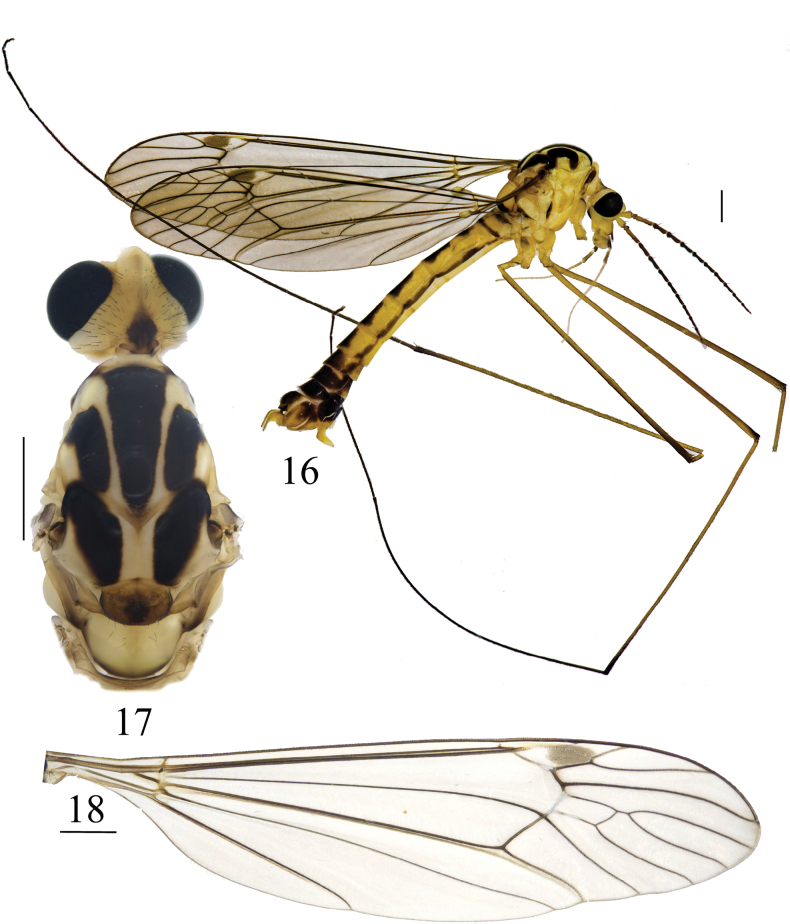
*Nephrotoma geniculata*, male. 16. Male habitus, lateral view; 17. Head and thorax, dorsal view; 18. Right wing. Scale bars: 1.0 mm.

***Head*** (Figs 16, 17). Mostly yellow, occipital marking drop-shaped, apically pale brown. Nasus dark brown. Rostrum brown. Head with black setae. Antenna 13-segmented, reaching the base of abdomen when bent backward; scape yellow, pedicel and first flagellar segment yellowish brown, the rest of segments brown. Verticils about twice shorter than the corresponding segment. Base of flagellar segments broader. Terminal segment of flagellum round. Palpi brownish, terminal segment pale, darkened at base.

***Thorax*** (Figs 16, 17). Mostly yellow. Pronotum yellow, black middorsally. Prescutum and presutural scutum with three longitudinal black stripes; lateral stripe apically bent outward. Postsutural scutum yellow in the middle, each lobe with large black spot. Scutellum brown, variegated with a round black stripe at the middle-lower part. Mediotergite with posterior margin blackened. Anepisternum and katepisternum basally each with a large brown spot. Anepimeron pale brown apically; katepimeron with small median dark yellow spot. Meron pale brown basally. Legs yellow. Coxae yellow, variegated with brown spots at the base. Apices of tibiae and femora dark brown, tarsal segments dark brown. Wing pale brown with a distinct dark brown pterostigma and macrotrichiae; pale brown shadow at vein *R4+5* and crossvein r-m; cell m1 petiolate (Fig. 18). Halter with stem brown; knob pale yellow with a dark brown margin (Fig. 16).

***Abdomen*** (Fig. 16). Mainly yellow. Abdominal tergites with an interrupted trapezoidal black stripe at the middle and two continuous black stripes on both sides; the median stripe broader than the lateral stripes. Abdominal segments 8–9 black.

**Figures 19–23.**
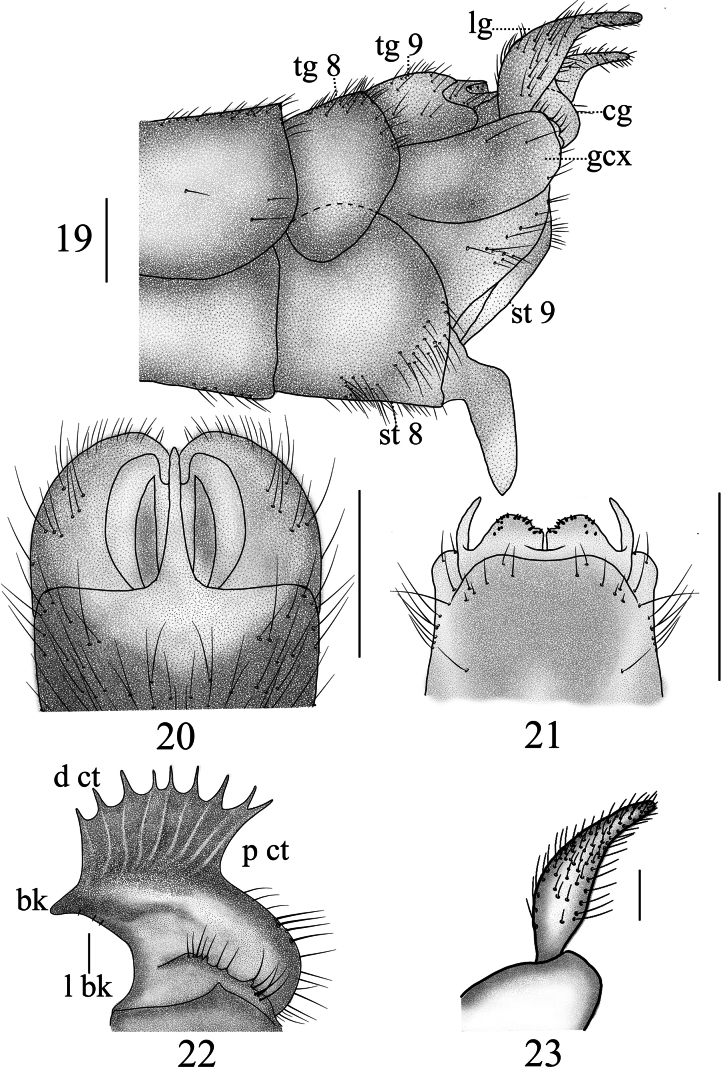
Male terminalia of *Nephrotoma geniculata*. 19. Hypopygium, lateral view; 20. Hypopygium, ventral view; 21. Tergite 9, dorsal view; 22. Clasper of gonostylus, lateral view; 23. Lobe of gonostylus, lateral view. Scale bars: 0.5 mm (19–21); 0.1 mm (22, 23).

**Figures 24–26.**
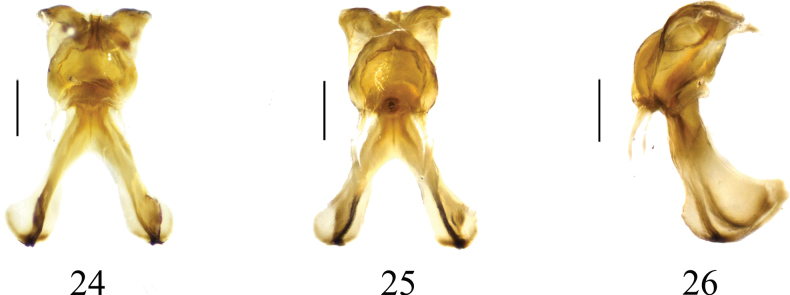
Semen pump of *Nephrotoma geniculata*. 24. Dorsal view; 25. Ventral view; 26. Lateral view. Scale bars: 0.1 mm.

***Hypopygium*** (Figs 19–23). Tergite 9 posteriorly strongly sclerotized with four projections: median projections broad, covered with short, tiny black spines, separated by narrow incision; lateral projections in the shape of narrow, inwardly curved horns; additional rounded projections on each side (Fig. 21). Sternite 8 posteriorly with a bent, flattened protuberance, narrowing downwards in lateral view; apex pointed in ventral view (Fig. 20). Sternite 9 posteriorly with sparse setae, and a U-notch sclerotized projection at the middle in ventral view (Fig. 20). Gonocoxite separated from sternite 9, nearly rectangular in shape with smooth margins (Fig. 19). Clasper of gonostylus with a large concavity at the base; beak short and obtuse; dorsal crest sclerotized with cristate projection; the margin extending from the beak to the lower beak, strongly sclerotized (Fig. 22). Lobe of gonostylus broadened at basal half, apical half narrowed (Figs 19, 23). Semen pump brownish yellow (Figs 24–26). Posterior immovable apodeme directed backward, tip inward; compressor apodeme flattened, fan-shaped, forming a 35° angle with the posterior immovable apodeme, with deep median incision. Anterior immovable apodeme triangular, with a sharp tip.

**Figures 27–29.**
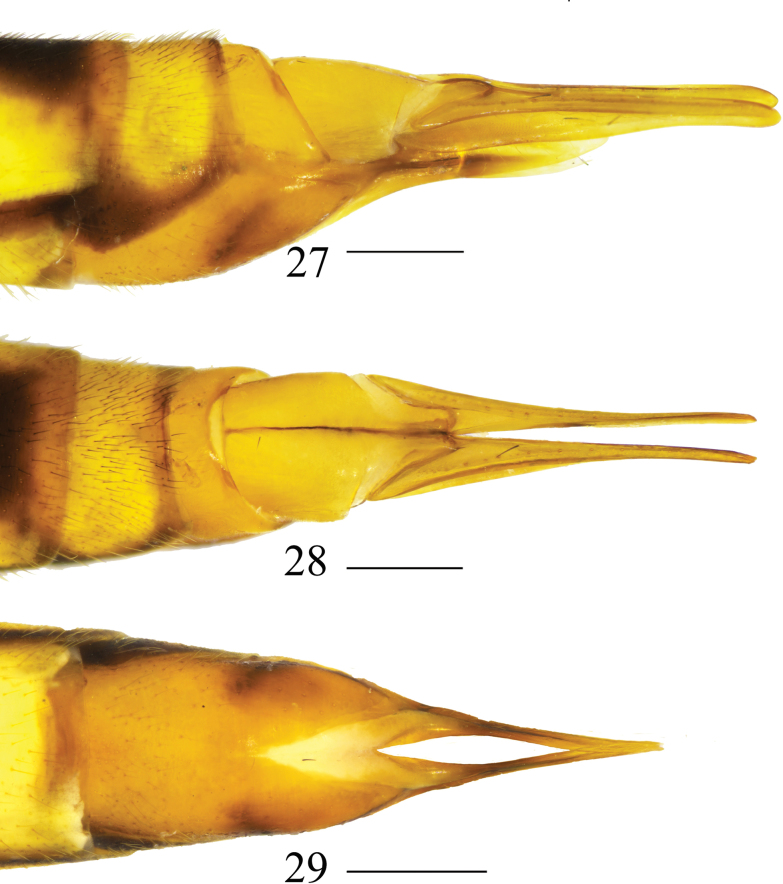
Female terminalia of *Nephrotoma geniculata*. 27. Ovipositor, lateral view; 28. Ovipositor, dorsal view; 29. Ovipositor, ventral view. Scale bars: 0.5 mm.

**Female.** Body length 15.4–16.7 mm, wing length 14.1–14.9 mm, antenna length 3.6–3.7 mm (*N* = 11). Resembles male in head, thorax and abdomen. Female antenna second pedicel yellow. Tergite 8 and 9 with red-brown margins, the rest yellow (Figs 27–29). Sternite 9 with red-brown round spots on the middle side.

***Ovipositor*** (Figs 27–29). Yellowish brown. Cercus narrowed toward tip (Figs 27, 28). Hypogynial valve curved downward apically (Figs 27, 29).

##### Elevation range in China.

Adults were collected at altitudes ranging from 2300 m to 2500 m.

##### Period of activity.

Adults are flying in June–July.

##### Distribution.

China (Guizhou; Inner Mongolia; Ningxia; Qinghai: Minle, Menyuan, Huzhu, Qilian; Sichuan).

##### Remarks.

*Nephrotoma geniculata* is similar to *N. parvirostra* Alexander, 1924, in the shape of tergite 9 and the lobe of the gonostylus. However, it can be distinguished by the following features: in *N. geniculata*, sternite 8 posteriorly exhibits a bent, flattened protuberance. In *N. parvirostra*, sternite 8 posteriorly lacks any prominent protuberance but Sternite 9 posteriorly possesses a lamellate protuberance (Oosterbroek 1985; Yang and Yang 1987a).

#### ﻿ Nephrotoma joneensis

Taxon classificationAnimaliaDipteraTipulidae

Yang & Yang, 1990

BE8CEDF0-CF01-5515-995B-C3972B51751B

Figs 30–32, 33–37, 38–40, 41–43

Nephrotoma joneensis Yang & Yang, 1990: 481.

##### Material examined.

China – Gansu Prov. • 1 ♂, 1 ♀; Zhuoni, Ganbuta; 2900 m a.s.l.; 16 Aug. 1980; F.S. Li leg.; CAU 491, 492. – Qinghai Prov. • 1 ♂; Minhe, Gushan Forest Farm; 2456 m a.s.l.; 36.0841°N, 102.7100°E; 29 Jun. 2019; X. Li leg.; CAU TN2304205 • 1 ♂; Minhe; Shenba Village; 2455 m a.s.l.; 36.2496°N, 102.5274°E; 29 Jun. 2019; Qilemoge leg.; CAU TN2304233 • 2 ♂♂, 2 ♀♀; Huzhu, Dongyuanguidaodongce; 2756 m a.s.l.; 36.7803°N, 102.1365°E; 1 Jul. 2019; Y. Liu leg.; CAU TN2304323 to TN2304326 • 2 ♂♂, 2 ♀♀; Huzhu, Tianmensi Temple; 3125 m a.s.l.; 36.7541°N, 102.2005°E; 1 Jul. 2019; Qilemoge leg.; CAU TN2304026 to TN2304029 • 2 ♂♂; Huzhu, Weibei Road; 2490 m a.s.l.; 36.0304°N, 102.3309°E; 3 Jul. 2019; Qilemoge leg.; CAU TN2304125, TN2304126 • 1 ♂, 1 ♀; Huzhu, Weibei Road; 3017 m a.s.l.; 37.0023°N, 102.1226°E; 18 Jul. 2023; X. Wang leg.; XJAU TN2309001 to TN 2309002 • 4 ♂♂; Menyuan, Sigou; 2526 m a.s.l.; 37.1470°N, 102.3749°E; 20 Jul. 2023; X. Wang leg.; XJAU TN2309064 to TN2309067 • 1 ♂, 1 ♀; Menyuan, Sanchakou; 2709 m a.s.l.; 37.2780°N, 102.1880°E; 21 Jul. 2023; X. Wang leg.; XJAU TN2309078 to TN2309079 • 2 ♂♂; Menyuan, Meihua Village; 3101 m a.s.l.; 37.2847°N, 102.1429°E; 22 Jul. 2023; X. Wang leg.; XJAU TN2309225 to TN2309226 • 1 ♀; Menyuan, Qihankaigou; 2684 m a.s.l.; 37.1587°N, 102.0324°E; 22 Jul. 2023; X. Wang leg.; XJAU TN2309227 • 4 ♂♂, 1 ♀; Menyuan, Heiyegou; 2863 m a.s.l.; 37.2527°N, 102.34224°E; 23 Jul. 2023; X. Wang leg.; XJAU TN2309235 to TN2309239 • 1 ♂; Menyuan, G569; 2580 m a.s.l.; 37.2334°N, 102.0173°E; 24 Jul. 2023; X. Wang leg.; XJAU TN2309280 • 12 ♂♂, 1 ♀; Menyuan, Laohugou; 3395 m a.s.l.; 37.5533°N, 101.5885°E; 26 Jul. 2023; X. Wang leg.; XJAU TN2309332 to TN2309344 • 12 ♂♂, 3 ♀♀; Qilian, Longkong Section; 3395 m a.s.l.; 38.0741°N, 100.6425°E; 27 Jul. 2023; X. Wang leg.; XJAU TN2310015 to TN2310029 • 2 ♂♂; Qilian, Binggou; 38.1333°N, 3075 m a.s.l.; 100.1678°E; 27 Jul. 2023; X. Wang leg.; light trap; XJAU TN2310013 to TN2310014 • 2 ♂♂, 2 ♀♀; Qilian, Qingyanggou; 3210 m a.s.l.; 38.1619°N, 100.4799°E; 28 Jul. 2023; X. Wang leg.; XJAU TN2310030 to TN2310033 • 1 ♂; Qilian, Qingyanggou; 3152 m a.s.l.; 38.1540°N, 100.3962°E; 28 Jul. 2023; X. Wang leg.; light trap; XJAU TN2310008 • 1 ♂, 1 ♀; Qilian, Yindonggou; 3211 m a.s.l.; 38.1619°N, 100.3922°E; 28 Jul. 2023; X. Wang leg.; XJAU TN2310203 to TN2310204 • 1 ♂, 2 ♀♀; Qilian, Zhamashidonggou; 3050 m a.s.l.; 38.1546°N, 100.0227°E; 29 Jul. 2023; X. Wang leg.; XJAU TN2310034 to TN2310036 • 10 ♂♂, 4 ♀♀; Qilian, Lujiaogou; 3311 m a.s.l.; 38.1132°N, 100.4744°E; 30 Jul. 2023; X. Wang leg.; light trap; XJAU TN2310216 to TN2310229 • 2 ♂♂, 2 ♀♀; Qilian, Lujiaogou; 3269 m a.s.l.; 38.1164°N, 100.4638°E; 30 Jul. 2023; X. Wang leg.; XJAU TN2310188 to TN2310191 • 1 ♂; Qilian, Lujiaogou; 2867 m a.s.l.; 38.1164°N, 100.3407°E; 30 Jul. 2023; X. Wang leg.; light trap; XJAU TN2310050 • 8 ♂♂, 7 ♀♀; Qilian, Binggou; 3410 m a.s.l.; 38.1259°N, 100.1283°E; 31 Jul. 2023; X. Wang leg.; XJAU TN2310110 to TN2310124 • 1 ♂, 3 ♀♀; Qilian, Yeniugou; 3300 m a.s.l.; 38.3753°N, 99.4473°E; 1 Aug. 2023; X. Wang leg.; XJAU TN2310139 to TN2310142 • 3 ♂♂, 1 ♀; Qilian, Youhulu; 3094 m a.s.l.; 38.2530°N, 99.7875°E; 1 Aug. 2023; X. Wang leg.; XJAU TN2310143 to TN2310146 • 1 ♀; Qilian, Baiyanggou; 2871 m a.s.l.; 38.2192°N, 100.2634°E; 2 Aug. 2023; X. Wang leg.; light trap; XJAU TN2310149 • 3 ♂♂, 1 ♀; Qilian, Huangcang Temple Water Conservancy Hub Project; 2579 m a.s.l.; 38.2499°N, 100.1836°E; 2 Aug. 2023; X. Wang leg.; light trap; XJAU TN2310165 to TN2310168.

##### Diagnosis.

Body black. Vertex with a tubercle and a dark-yellow spot at the posterior-lateral margin. Antenna reaching the base of abdomen when bent backward; base of flagellar segments enlarged starting from the second segment. Prescutum and presutural scutum median and lateral stripes fused. Tergite 9 posteriorly with horned, inclined projections and median V-shaped notch. Sternite 8 posteriorly with a median, apically bifid appendage. Clasper of gonostylus with dorsal crest bare, lower break horn shaped.

##### Redescription.

**Male.** Body length 11.1–14.3 mm, wing length 8.9–11.6 mm, antenna length 3.5–5.7 mm (*N* = 81). Present description based on immersed specimens.

**Figures 30–32.**
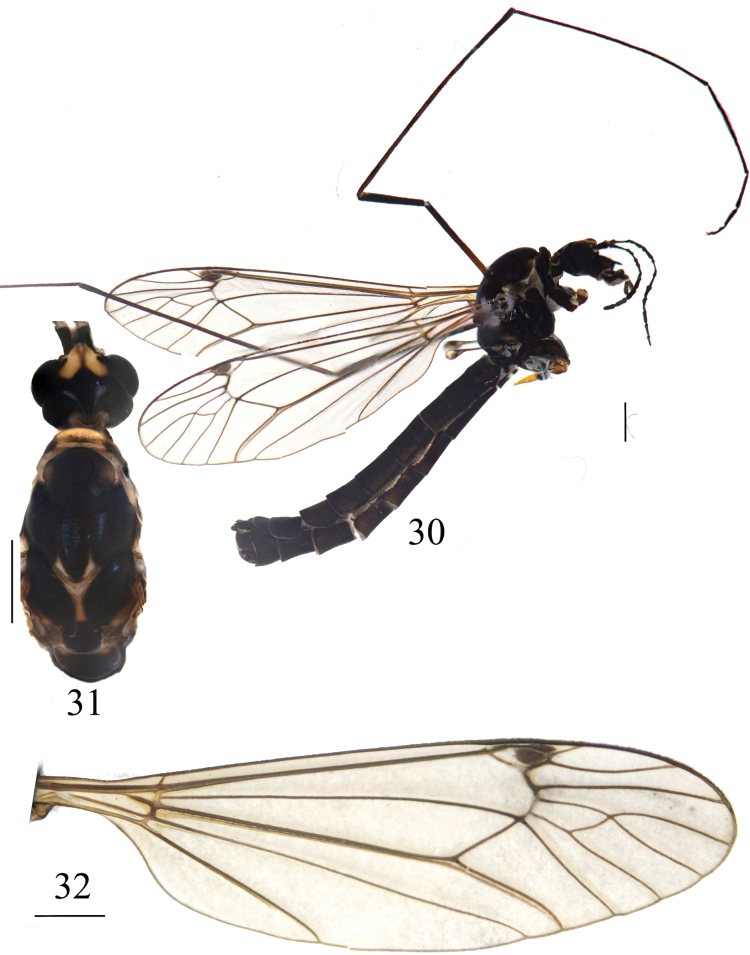
*Nephrotoma joneensis*, male. 30. Male habitus, lateral view; 31. Head and thorax, dorsal view; 32. Right wing. Scale bars: 1.0 mm.

***Head*** (Figs 30, 31). Mostly black, vertex with a tubercle, a petaloid dark yellow spot at posterior-lateral margin. Nasus obvious. Head with black setae. Antenna 13-segmented, reaching the base of abdomen when bent backward; scape brown, with apical third brown, pedicel and flagellar segments dark brown. Base of the flagellum segments enlarged starting from the second segment. Verticils about twice shorter than the corresponding segment. Terminal segment of flagellum round. Palpi blackened, terminal segment pale, darken at base.

***Thorax*** (Figs 30, 31). Mostly black. Pronotum dark yellow, black middorsally. Prescutum and presutural scutum with three fused longitudinal black stripes; median stripes apically dark brown. Postsutural scutum brown in the middle, each lobe with large black spot. Scutellum black. Mediotergite dark yellow with two dark-brown spots apically. Legs black. Coxae black. Apices of tibiae brown, apices of femora dark brown, tarsal segments black. Wing pale brown, with a distinct black pterostigma; black shadow at vein *R4+5* and crossvein r-m; cell m1 petiolate (Fig. 32). Halter with stem black; knob pale black variegated with a dark-brown spot (Fig. 30).

***Abdomen*** (Fig. 30). Entirely black.

**Figures 33–37.**
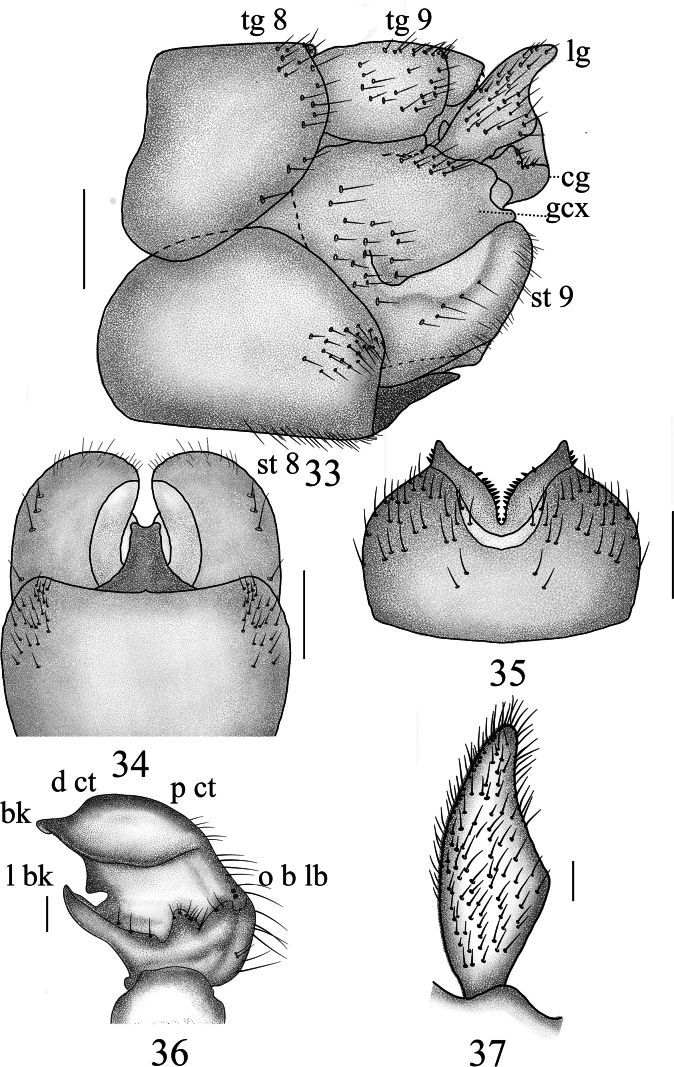
Male terminalia of *Nephrotoma joneensis*. 33. Hypopygium, lateral view; 34. Hypopygium, ventral view; 35. Tergite 9, dorsal view; 36. Clasper of gonostylus, lateral view; 37. Lobe of gonostylus, lateral view. Scale bars: 0.5 mm (33–35); 0.1 mm (36, 37).

**Figures 38–40.**
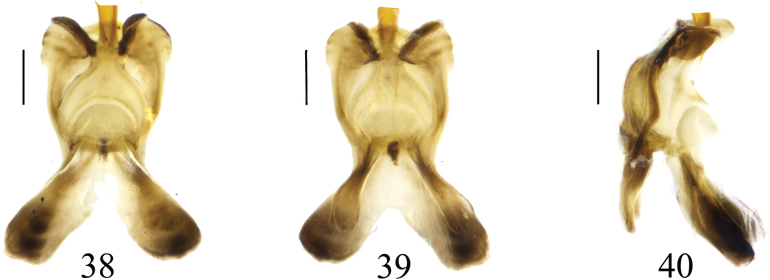
Semen pump of *Nephrotoma joneensis*. 38. Dorsal view; 39. Ventral view; 40. Lateral view. Scale bars: 0.1 mm.

***Hypopygium*** (Figs 33–37). Tergite 9 posteriorly with horned, inclined projections, and a narrow, median V-shaped notch. Median region with a white crescent membrane (Fig. 35). Sternite 8 posteriorly with apically bifid appendage, strongly sclerotized at the middle (Fig. 34). Sternite 9 posteriorly with sparse setae, the median U-notch sclerotized at the middle in ventral view (Figs 33, 34). Gonocoxite separated from sternite 9, apically bearing shallow incision (Fig. 33). Clasper of gonostylus with a big concavity at the middle; beak obtuse; dorsal crest bare; outer basal lobe splitted into two short plates; lower beak with horned protuberance; the margin extending from the beak to the lower beak, strongly sclerotized; one U-notch between the beak and the lower beak (Fig. 36). Lobe of gonostylus broadened at the middle, rest of sclerite tapering towards apex. (Figs 37). Semen pump yellowish (Figs 38–40). Posterior immovable apodeme leaf-shaped directed backward, tip inward. Compressor apodeme dark brown fan-shaped, forming a 30° angle with the posterior immovable apodeme. Anterior immovable apodeme with inner margin black, narrower than the compressor apodeme.

**Figures 41–43.**
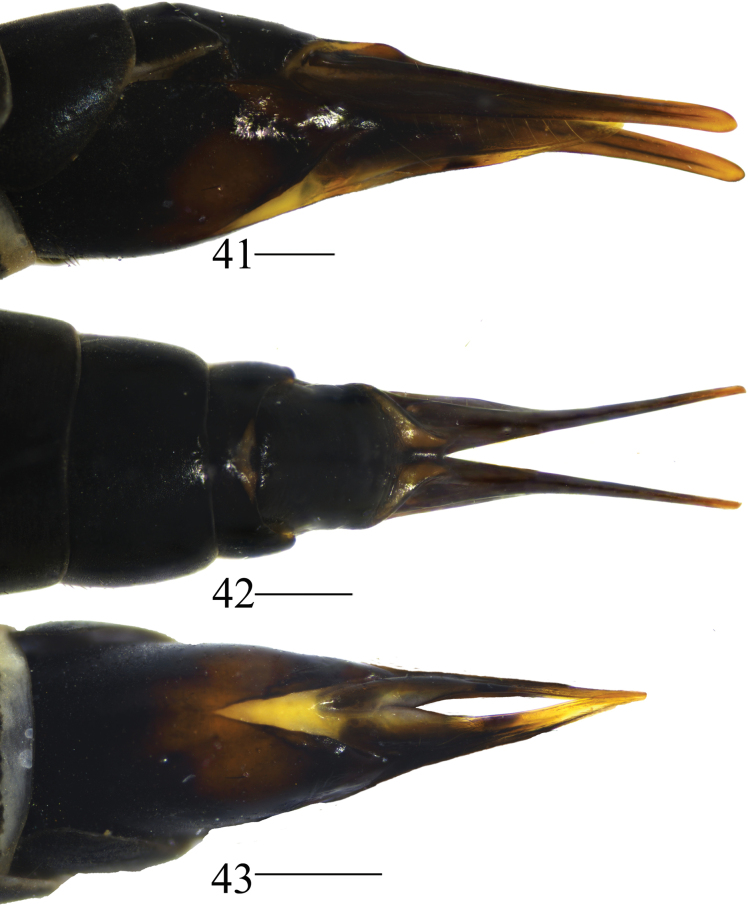
Female terminalia of *Nephrotoma joneensis*. 41. Ovipositor, lateral view; 42. Ovipositor, dorsal view; 43. Ovipositor, ventral view. Scale bars: 0.5 mm.

**Female.** Body length 14.3–18.4 mm, wing length 9.7–14.2 mm, antenna length 2.3–3.7 mm (*N* = 37). Resembles male in head, thorax and abdomen. Abdominal segments of females with dorsal plates shallowly tinged with yellow near lateral margins. Tergite 8, tergite 9 black, tergite 10 dark brown (Figs 41–43).

***Ovipositor*** (Figs 41–43). Black. Cercus narrowed toward tip (Figs 41, 42). Hypogynial valve curved downward apically (Figs 41, 43). Both sides of hypovalva yellow and black at the middle (Fig. 43).

##### Elevation range in China.

Adults were collected at altitudes ranging from 2455 m to 3410 m.

##### Period of activity.

Adults are flying in June–August.

##### Distribution.

China (Gansu, Qinghai: Huzhu, Menyuan, Qilian).

##### Remarks.

*Nephrotoma joneensis* is similar to *N. basiflava* Alexander, 1925 in body color. However, it can be distinguished by the following features: in *N. joneensis*, tergite 9 has two projections; sternite 8 posteriorly has a bifid appendage; the overall body size is larger. In *N. basiflava*, tergite 9 has four projections; sternite 8 posteriorly is flat at the middle, without an appendage; the overall body size is smaller. (Yang and Yang 1991, 2002).

#### ﻿ Nephrotoma ligulata

Taxon classificationAnimaliaDipteraTipulidae

Alexander, 1925

D68B5F28-4295-517A-8322-93E552D756E7

Figs 44–46, 47–51, 52–54, 55–57

Nephrotoma ligulata Alexander, 1925: 402.
Pachyrhina pygmaea Dodonov, 1926: 106 (synonymy).

##### Material examined.

China – Xinjiang U.A.R. • 5 ♂♂, 3 ♀♀; Xinjiang Production and Construction Corps, 165^th^ Regiment, 4^th^ Company; 1457 m a.s.l.; 43.9440°N, 84.4977°E; 2 Aug. 2017; B. Zhang leg.; CAU TN2306266 to TN2306273 • 12 ♂♂, 5 ♀♀; Xinjiang Production and Construction Corps, 165^th^ Regiment, 4^th^ Company; 1457 m a.s.l.; 46.8554°N, 84.3583°E; 2 Aug. 2017; B. Zhang leg.; CAU TN2306280 to TN2306296 • 5 ♂♂; Xinjiang Production and Construction Corps, 165^th^ Regiment, 4^th^ Company; 1301 m a.s.l.; 46.9486°N, 84.5622°E; 2 Aug. 2017; J.L. Ren leg.; CAU TN2306322 to TN2306326 • 2 ♂♂, 2 ♀♀; Yili, Nalati; 1813 m a.s.l.; 42.2175°N, 84.3155°E; 15 Jul. 2023; J.L. Ren leg.; CAU TN2310239 to TN2310242.

##### Diagnosis.

Body yellow. Occipital marking peach-shaped, dark brown. Antenna reaching the base of abdomen when bent backward. Wing pale brown, cell m1 mostly sessile, some specimens petiolate. Tergite 9 posteriorly with broad, flat projections, covered with short, tiny black spines, and separated by narrow incision. Sternite 8 posteriorly with a short, bifid appendage. Clasper of gonostylus with posterior crest bearing a triangular projection and sparse setae.

##### Redescription.

**Male.** Body length 9.9–11.3 mm, wing length 9.9–10.3 mm, antenna length 3.3–4.9 mm (*N* = 24). Present description based on specimens preserved in alcohol.

**Figures 44–46.**
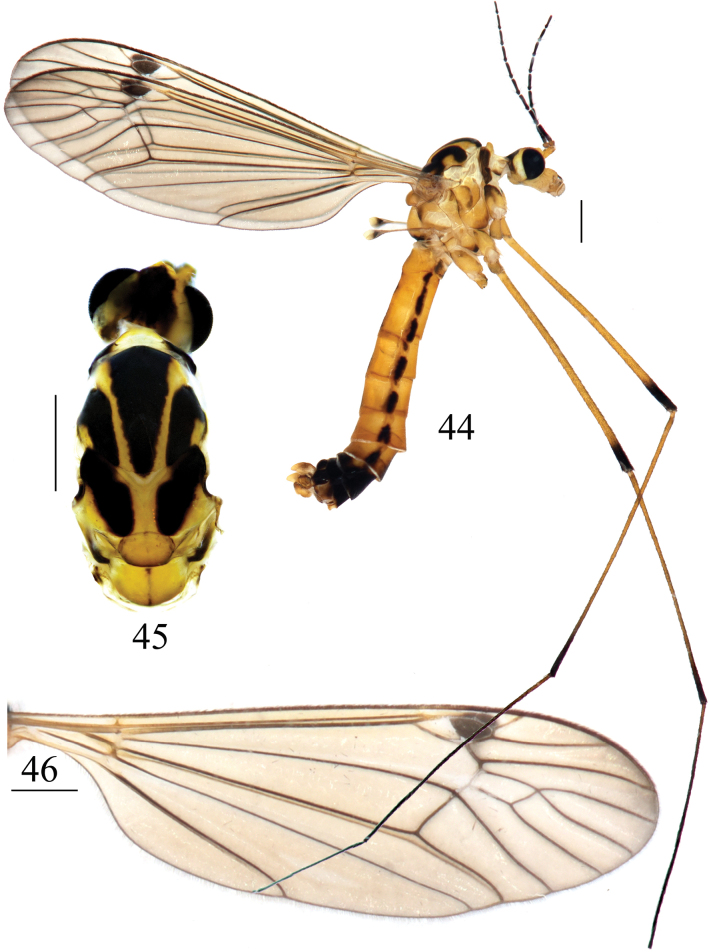
*Nephrotoma ligulata*, male. 44. Male habitus, lateral view; 45. Head and thorax, dorsal view; 46. Right wing. Scale bars: 1.0 mm.

***Head*** (Figs 44, 45). Mostly yellow. Occipital marking peach-shaped, dark brown. Nasus dark brown. Rostrum brown. Head with brown setae. Antenna 13-segmented, reaching the base of abdomen when bent backward; scape yellow, with apical third brown, pedicel and flagellar segments dark brown. Length of verticils reaching almost 3/5 length of the corresponding segment. Terminal segment of flagellum round. Palpi brownish, terminal segment pale, darkened at base.

***Thorax*** (Figs 44, 45). Mostly yellow. Pronotum black, pale yellow middorsally. Prescutum and presutural sctum with three longitudinal black stripes; lateral stripe apically bent outward. Postsutural scutum yellow in the middle, each lobe with large black spot. Scutellum yellow with brown margins. Mediotergite yellow with thin black median stripe. Anepisternum and katepisternum basally each with a large black spot. Anepimeron blackened apically; katepimeron with small brown median spot. Meron blackened basally. Legs yellow. Coxae yellow variegated with dark brown spots at the base. Apices of tibiae, femora, and tarsi dark brown. Wing pale brown with a distinct dark brown pterostigma; brown shadow at vein *R4+5* and crossvein r-m; cell m1 mostly sessile, some specimens petiolate (Fig. 46). Halter with stem brown; knob pale yellow with a dark-brown margin and base (Fig. 44).

***Abdomen*** (Fig. 44). Mostly yellow. Abdominal tergites on segments 1–4 with one median longitudinal stripe at the posterior margin and two interrupted stripes on both sides. Abdominal sternites 5–7 with an interrupted longitudinal stripe. Abdominal segments 7–9 black.

**Figures 47–51.**
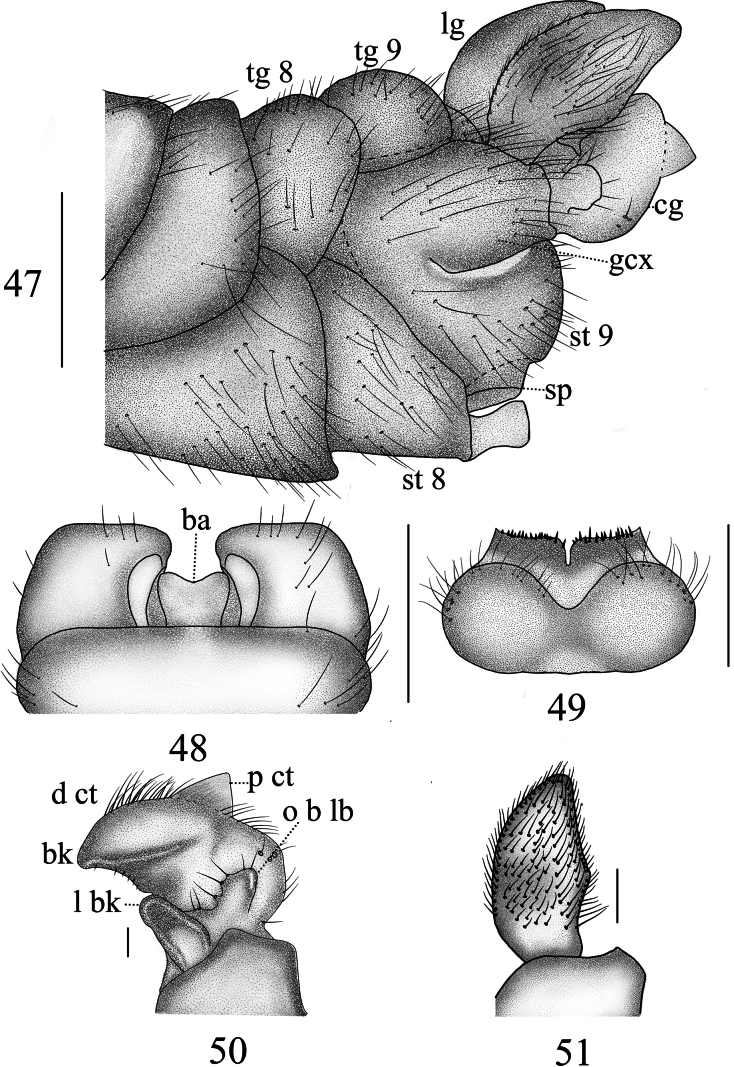
Male terminalia of *Nephrotoma ligulata*. 47. Hypopygium, lateral view; 48. Hypopygium, ventral view; 49. Tergite 9, dorsal view; 50. Clasper of gonostylus, lateral view; 51. Lobe of gonostylus, lateral view. Scale bars: 0.5 mm (47–49); 0.1 mm (50, 51).

**Figures 52–54.**
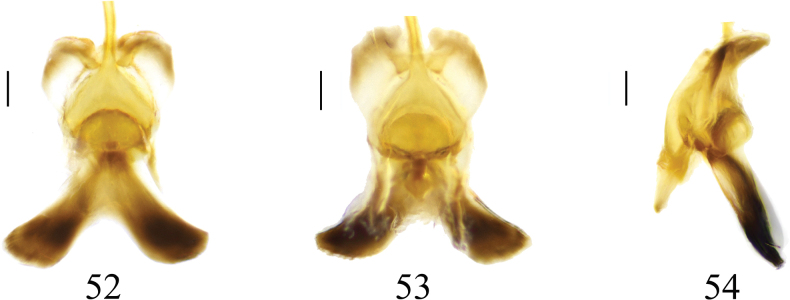
Semen pump of *Nephrotoma ligulata*. 52. Dorsal view; 53. Ventral view; 54. Lateral view. Scale bars: 0.1 mm.

***Hypopygium*** (Figs 47–51). Tergite 9 posteriorly with broad, flat projections, covered with short, tiny black spines, separated by narrow median incision (Fig. 49). Sternite 8 posteriorly with a short, broad, apically bifid appendage (Fig. 48). Sternite 9 posteriorly with sparse setae, and a sclerotized projection at the middle (Figs 47, 48). Gonocoxite separated from sternte 9, nearly rectangular in shape (Fig. 47). Clasper of gonostylus with a large concavity at the base; beak obtuse; posterior crest with a triangular projection and sparse setae; outer basal lobe short, nearly oval; lower beak with nearly oval protuberance; the margin extending from the beak to the lower beak, strongly sclerotized (Fig. 50). Lobe of gonostylus broadens from base to middle, tapers to apex (Figs 47, 51). Semen pump mostly yellow (Figs 52–54). Posterior immovable apodeme tapered, directed backward. Compressor apodeme fan-shaped, forming a 60° angle with the posterior immovable apodeme. Anterior immovable apodeme with a brown margin, narrower than the compressor apodeme.

**Figures 55–57.**
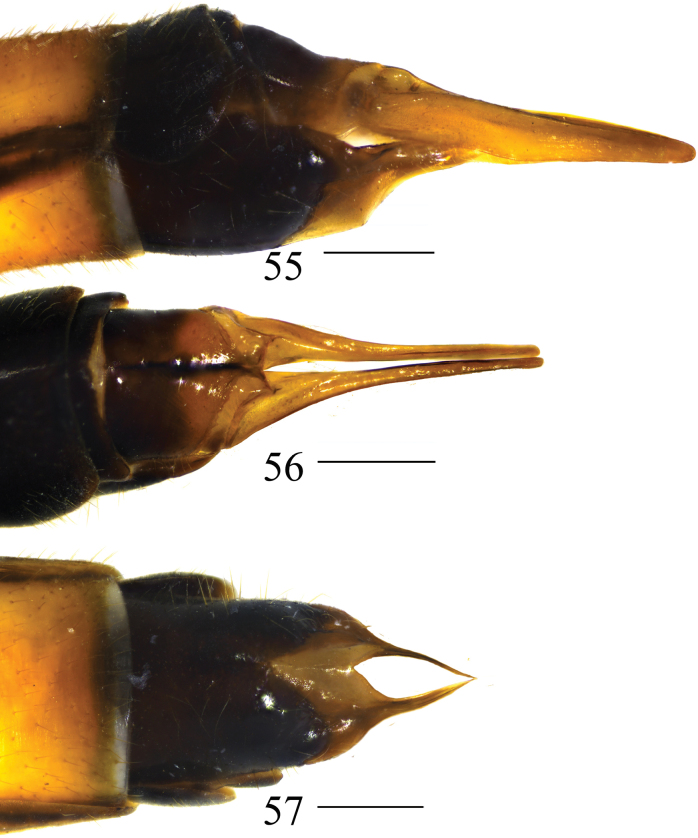
Female terminalia of *Nephrotoma ligulata*. 55. Ovipositor, lateral view; 56. Ovipositor, dorsal view; 57. Ovipositor, ventral view. Scale bars: 0.5 mm.

**Female.** Body length 14.4–15.5 mm, wing length 10.6–12.6 mm, antenna length 2.9 mm (*N* = 10). Resembles male in head, thorax and abdomen. Sternite 7 unmarked at the middle. Tergite 8, tergite 9, sternite 8 and basal region of tergite 10 black (Figs 55–57).

***Ovipositor*** (Figs 55–57). Reddish brown. Cercus narrowed toward tip (Figs 55, 56). Hypogynial valve curved downward at apex (Figs 55, 57).

##### Elevation range in China.

Adults were collected at altitudes ranging from 1300 m to 1810 m.

##### Period of activity.

Adults are flying in July–August.

##### Distribution.

Afghanistan, China (Xinjiang), India (north), Kyrgyzstan, Mongolia, Russia, Turkmenistan, Tajikistan, Uzbekistan.

##### Remarks.

*Nephrotoma ligulata* is similar to *N. biformis* Alexander, 1935, particularly in that both have a bifurcated sternite 8. However, it can be distinguished by the following features: in *N. ligulata*, the clasper of the gonostylus has a posterior crest with a triangular projection and sparse setae; tergite 9 posteriorly features a seam at the middle. In *N. biformis*, the clasper of the gonostylus has a smooth posterior crest without any prominent projections and sparse setae; tergite 9 posteriorly features a V-notch at the middle (Alexander 1935b; Mannheims 1967; Liu 2011).

#### ﻿ Nephrotoma lunulicornis

Taxon classificationAnimaliaDipteraTipulidae

(Schummel, 1833)

2CBB1C86-1FA2-538A-9AC0-DB02122F6F34

Figs 58–60, 61–65, 66–68

Tipula lunulicornis Schummel, 1833: 107.
Tipula picta Meigen, 1838: 35 (synonymy).

##### Material examined.

China – Qinghai Prov. • 5 ♂♂; Menyuan, Sanchakou; 2709 m a.s.l.; 37.2780°N, 102.1880°E; 21 Jul. 2023; X. Wang leg.; XJAU TN2309210 to TN2309214.

##### Diagnosis.

Body yellow. Occipital marking spine shaped. Vertex with a dark-brown round spot. Vein *R4+5* and crossvein r-m with dark-brown cloud-shaped shadow. Abdominal tergites with one interrupted longitudinal median stripe. Tergite 9 posteriorly with two projections; outer projection finger-shaped and covered with short, tiny black spines. Sternite 8 posteriorly with setae and a deep V-notch. Sternite 9 with a horned appendage directed ventrally in lateral view. Clasper of gonostylus with dorsal crest with margin forming flattened projection.

##### Redescription.

**Male.** Body length 12.5–14.6 mm, wing length 13.6–15.2 mm, antenna length 4.7–5.4 mm (*N* = 5). Present description based on specimens preserved in alcohol.

**Figures 58–60.**
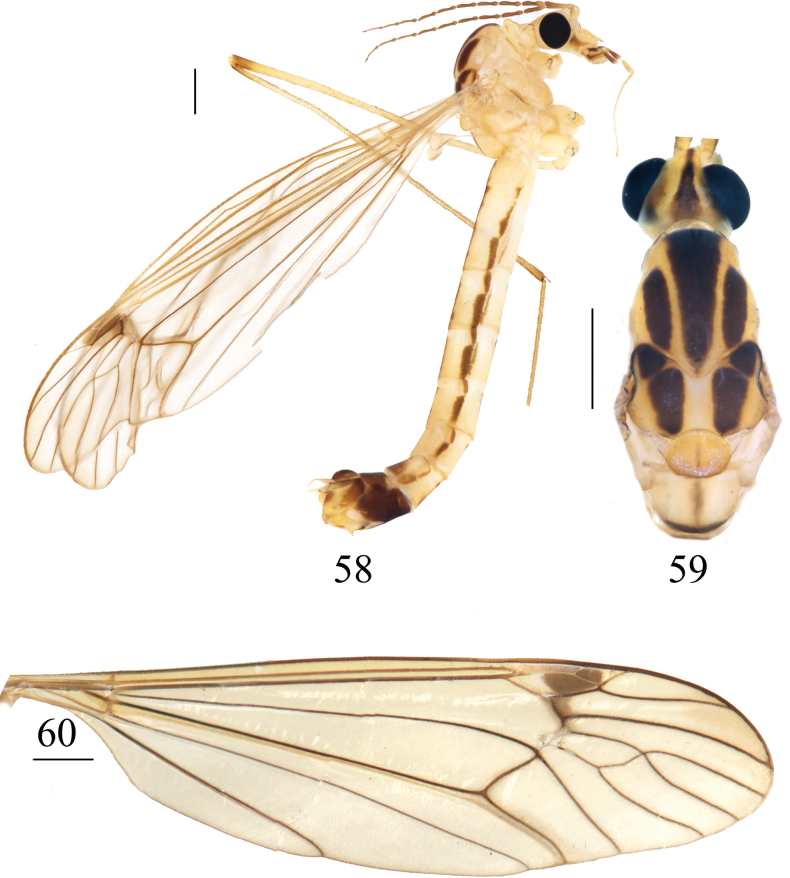
*Nephrotoma lunulicornis*, male. 58. Male habitus, lateral view; 59. Head and thorax, dorsal view; 60. Right wing. Scale bars: 1.0 mm.

***Head*** (Figs 58, 59). Mostly yellow. Occipital marking spine-shaped, with a small, round, brown spot anteriorly and an oval, brown patch posteriorly. Nasus dark yellow. Rostrum dark yellow. Head with black setae. Antenna 13-segmented, reaching the base of abdomen when bent backward; scape yellow except pale brown apex, pedicel and flagellum light brown. Verticils about the same length as the corresponding segments. Base of flagellar segments broader with the distal end also widened ventrally. Terminal segment of flagellum round. Palpi brownish, terminal segment pale, darkened at base.

***Thorax*** (Figs 58, 59). Mostly yellow. Pronotum yellow, brown posterior margin. Prescutum and presutural scutum with three longitudinal dark-brown stripes; lateral stripe apically straight. Postsutural scutum yellow in the middle, each lobe with two large black spots. Scutellum yellow, with brown line. Mediotergite with brown median stripe. Pleura light yellow. Legs yellow. Coxae yellow, variegated with brown spots at the base. Apices of tibiae and femora pale brown, tarsal segments dark brown. Wing pale brown with a distinct dark-brown pterostigma and macrotrichiae; dark-brown cloud-shaped shadow at vein *R4+5* and crossvein r-m; cell m1 mostly sessile (Fig. 60). Halter with stem brown; knob pale yellow with a dark-brown margin (Fig. 58).

***Abdomen*** (Fig. 58). Mainly yellow. Abdominal tergites with three dark-brown longitudinal stripes; the median stripe broader than the lateral stripes. Abdominal sternites with interrupted longitudinal stripe. Abdominal segments 8 and 9 black.

**Figures 61–65.**
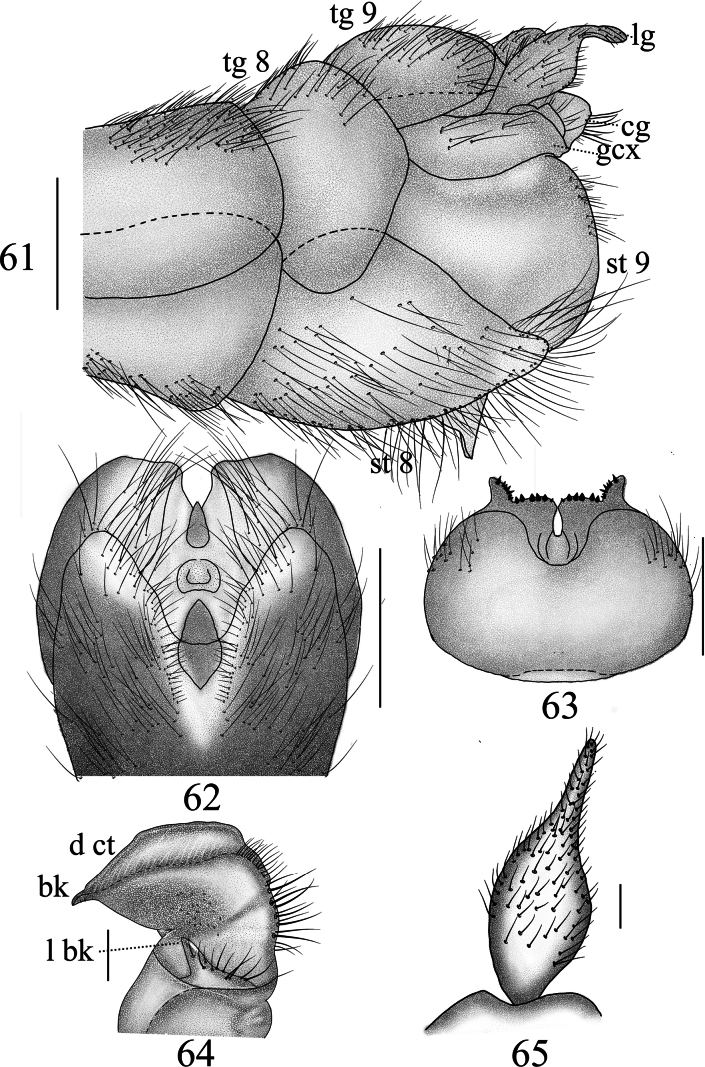
Male terminalia of *Nephrotoma lunulicornis*. 61. Hypopygium, lateral view; 62. Hypopygium, ventral view; 63. Tergite 9, dorsal view; 64. Clasper of gonostylus, lateral view; 65. Lobe of gonostylus, lateral view. Scale bars: 0.5 mm (61–63); 0.1 mm (64, 65).

**Figures 66–68.**
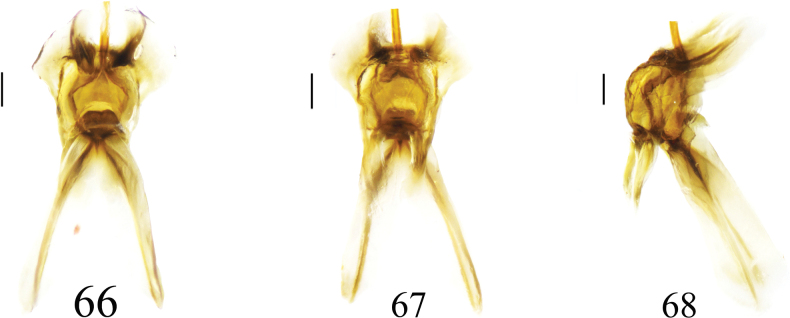
Semen pump of *Nephrotoma lunulicornis*. 66. Dorsal view; 67. Ventral view; 68. Lateral view. Scale bars: 0.1 mm.

***Hypopygium*** (Figs 61–65). Tergite 9 posteriorly strongly sclerotized with four projections: median projections broad, covered with short, tiny black spines, separated by narrow incision; lateral projections in the shape of finger-shaped, outwardly curved horns (Fig. 63). Sternite 8 posteriorly with setae on each side and a deep V-notch at the middle (Fig. 62). Sternite 9 with a horned appendage directed ventrally in lateral view (Fig. 62). Gonocoxite separated from sternite 9, having shape of nearly narrow rectangular (Fig. 61). Clasper of gonostylus with a large concavity at the base; beak long and acute; dorsal crest with margin forming flattened projection, posterior margin densely covered with golden bristles; lower beak with horned protuberance; the margin extending from the beak to the lower beak, strongly sclerotized (Fig. 64). Lobe of gonostylus broadened at basal half, apical half narrowed (Figs 61, 65). Semen pump dark yellow (Figs 66–68). Posterior immovable apodeme directed backward, tip inward. Compressor apodeme flattened, fan-shaped, forming a 40° angle with the posterior immovable apodeme, with deep median incision. Anterior immovable apodeme fan-shaped, broader than the compressor apodeme.

**Female.** Unknown.

##### Elevation range in China.

Adults were collected at altitudes of 2700 m.

##### Period of activity.

Adults were collected only in late July.

##### Distribution.

Andorra, Austria, Belarus, Belgium, Bosnia-Herzegovina, Bulgaria, China (Qinghai: Menyuan; Fig. 1), Croatia, Czech Republic, Denmark, Estonia, Finland, France, Germany, Great Britain, Hungary, Ireland, Italy (north), Latvia, Liechtenstein, Lithuania, Luxembourg, Montenegro, Netherlands, North Macedonia, Norway, Poland, Romania, Serbia, Slovakia, Slovenia, Sweden (south), Switzerland, Ukraine; Russia, Kazakhstan (east); Mongolia.

##### Remarks.

This is the first report of this species from China. *Nephrotoma lunulicornis* is similar to *N. laticrista* Alexander, 1925 based on the shape of tergite 9, sternite 8, and the lobe of the gonostylus. However, it can be distinguished by the following features: in *N. lunulicornis*, sternite 9 has a sharp, horned appendage directed ventrally in lateral view; the clasper of gonostylus has a smooth, prominent dorsal crest and densely covered with golden bristles. In *N. laticrista*, sternite 9 has a blunt, rounded horned appendage directed ventrally in lateral view; the clasper of gonostylus has a dorsal crest with a prominent projection that includes an indentation or depression (Tangelder 1984).

#### ﻿ Nephrotoma palacea sp. nov.

Taxon classificationAnimaliaDipteraTipulidae

57F41812-3A7D-5D1D-9C53-B6089C207843

https://zoobank.org/BA7CB6ED-32A3-44B0-BEC4-FB59FDE47885

Figs 69–71, 72–76, 77–79

##### Type material.

***Holotype*.** China – Xinjiang U.A.R. • ♂; Qinghe; Huahaizi; 2625 m a.s.l.; 46.4800°N, 90.5200°E; 14 Jul. 2016; J.L. Ren leg.; CAU TN2306185.

***Paratypes*.** China – Xinjiang U.A.R. • 5 ♂♂; same data as for holotype; CAU TN2306186 to TN2306190.

##### Diagnosis.

Body black. Vertex with a tubercle, a dark-yellow spot at the posterior-lateral margin. Antenna reaching the base of wing when bent backward. Wing with brown shadow at vein *R4+5* and crossvein r-m; cell m1 mostly sessile in most specimens. Tergite 9 posteriorly with horned projections, covered with black spines. Sternite 8 posteriorly with a horned, hairy, and sclerotized appendage that directed ventrally in lateral view. Clasper of gonostylus with long, straight, and obtuse beak; lower beak with spade-shaped protuberance; one U-notch between the beak and lower beak.

##### Description.

**Male.** Body length 9.7–11.6 mm, wing length 9.6–11.7 mm, antenna length 3.3–3.6 mm (*N* = 6).

**Figures 69–71.**
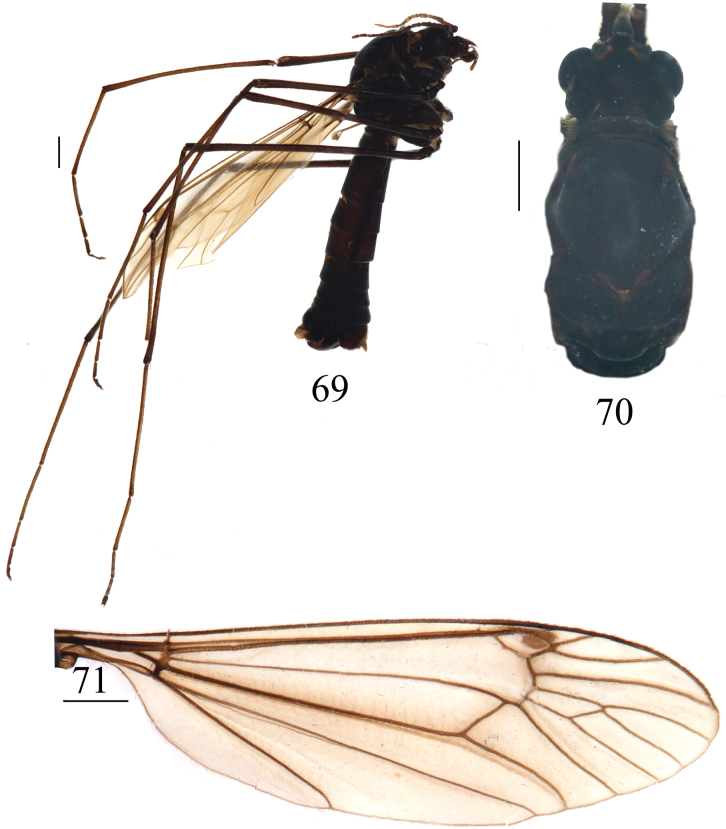
*Nephrotoma palacea* sp. nov., male. 69. Male habitus, lateral view; 70. Head and thorax, dorsal view; 71. Right wing. Scale bars: 1.0 mm.

***Head*** (Figs 69, 70). Mostly black, vertex with a tubercle, a dark-yellow spot at the posterior-lateral margin of a tubercle. Nasus obvious. Occiput postero-laterally with grey setae. Head with black setae. Antenna 13-segmented, reaching the base of wing when bent backward; scape short, dark brown, with apical third black, pedicel and flagellar segments black; flagellar segments except of the first one, enlarged at base. Length of verticils reaching almost 3/5 length of the corresponding segment. Terminal segment of flagellum round. Palpi black, terminal segment pale, darkened at base.

***Thorax*** (Figs 69, 70). Entirely black. Legs black. Coxae black. Middle portions of tibiae and femora dark brown, tarsal segments black. Wing dark brown, with a distinct dark-brown pterostigma and macrotrichiae; brown shadow at vein *R4+5* and crossvein r-m; cell m1 mostly sessile, some specimens petiolate (Fig. 71). Halter with stem black; knob grey variegated with a brown spot (Fig. 69).

***Abdomen*** (Fig. 69). Entirely black.

**Figures 72–76.**
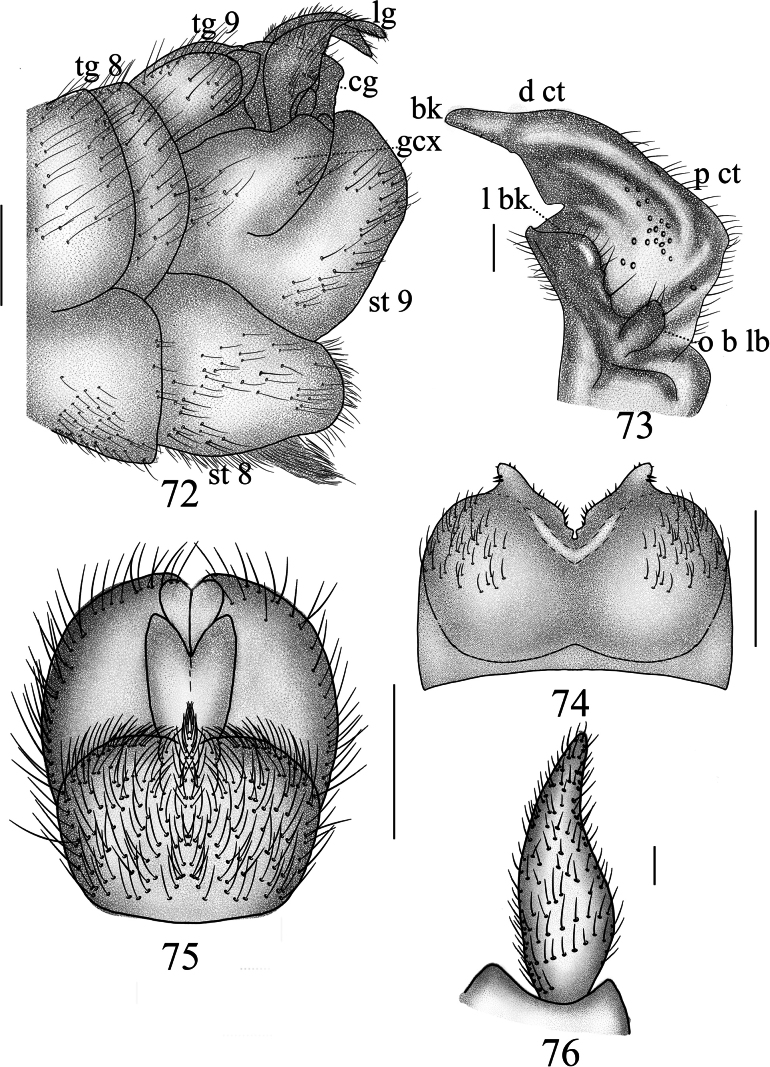
*Nephrotoma palacea* sp. nov., male. 72. Hypopygium, lateral view; 73. Clasper of gonostylus, lateral view; 74. Tergite 9, dorsal view; 75. Hypopygium, ventral view; 76. Lobe of gonostylus, lateral view. Scale bars: 0.5 mm (72, 74, 75); 0.1 mm (73, 76).

***Hypopygium*** (Figs 72–76). Tergite 9 posteriorly with horned projections, covered with black spines and separated by broad median V-shaped notch. Median region with a brown crescent membrane (Fig. 74). Sternite 8 posteriorly covered densely with setae, sunken at the middle in ventral view, and with a horned, hairy, and sclerotized appendage that directed ventrally in lateral view (Figs 72, 75). Sternite 9 posteriorly with sparse setae (Fig. 75). Gonocoxite separated from sternite 9, apically bearing a gradually tapering narrowness (Fig. 72). Lobe of gonostylus broadens from base to middle, tapers to apex (Figs 72, 76). Clasper of gonostylus with a large concavity at the middle; beak long, straight, and obtuse; dorsal crest posteriorly with strongly sclerotized projection and sparse setae; outer basal lobe in the shape of oval process, sclerotized margin, sparsely covered with setae; lower beak with spade-shaped protuberance; the margin extending from the beak to the lower beak, strongly sclerotized.

**Figures 77–79.**
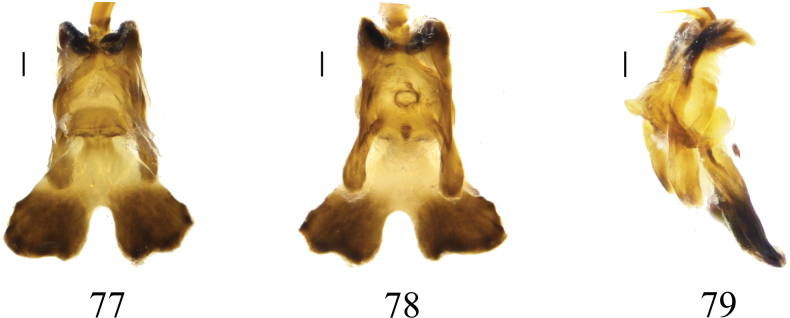
Semen pump of *Nephrotoma palacea* sp. nov. 77. Dorsal view; 78. Ventral view; 79. Lateral view. Scale bars: 0.1 mm.

Semen pump dark brown (Figs 77–79). Posterior immovable apodeme short, rod-like, directed backward, tip inward. Compressor apodeme flattened, apically with U-shaped incision forming two, distally darkened lobes. Anterior immovable apodeme with a strongly sclerotized margin, nearly triangular.

**Female.** Unknown.

##### Elevation range in China.

Adults were collected at altitudes of 2625 m.

##### Period of activity.

Adults were collected only in mid-July.

##### Distribution.

China, Xinjiang (Fig. 1).

##### Etymology.

The specific name (from Latin *palaceus*; adj., meaning “spade-shaped”) refers to spade-shaped protuberance at the lower beak of the clasper of gonostylus.

##### Remarks.

*Nephrotoma palacea* sp. nov. is similar to *N. erebus* Alexander, 1922, in habitus and based on the shape of sternite 8 and lobe of gonostylus. However, it can be distinguished by the following features: in *N. palacea*, the projections of tergite 9 are entirely covered with spines; the lower beak of the clasper of the gonostylus has a spade-shaped protuberance and is strongly sclerotized. In *N. erebus*, the projections of tergite 9 are covered with spines only at the apices; the lower beak of the clasper of the gonostylus has a lamellate protuberance and is strongly sclerotized only along the margin (Savchenko 1957, 1973). Additionally, discrepancies were found between Alexander’s original description and the characteristics of the type specimen. For example, the feature mentioned in the original text, “the yellow margin on sternites 4–6” (Alexander 1922), was not observed in the type specimen.

## Supplementary Material

XML Treatment for Nephrotoma furvligulata

XML Treatment for Nephrotoma geniculata

XML Treatment for Nephrotoma joneensis

XML Treatment for Nephrotoma ligulata

XML Treatment for Nephrotoma lunulicornis

XML Treatment for Nephrotoma palacea

## ﻿Additional information

### Use of AI

No use of AI was reported.

### Data availability

All of the data that support the findings of this study are available in the main text.
